# Leveraging pre-vaccination antibody titres across multiple influenza H3N2 variants to forecast the post-vaccination response

**DOI:** 10.1016/j.ebiom.2025.105744

**Published:** 2025-05-26

**Authors:** Hannah Stacey, Michael A. Carlock, James D. Allen, Hannah B. Hanley, Shane Crotty, Ted M. Ross, Tal Einav

**Affiliations:** a Center for Vaccine Innovation, La Jolla Institute for Immunology, La Jolla, CA 92037, USA; b Center for Vaccines and Immunology, University of Georgia, Athens, GA 30602, USA; c Department of Infectious Diseases, University of Georgia, Athens, GA 30602, USA; d Florida Research and Innovation Center, Cleveland Clinic, Port Saint Lucie, FL 34987, USA; e Department of Medicine, University of California San Diego, La Jolla, CA 92037, USA; f Department of Infection Biology, Lehner Research Institute, Cleveland Clinic, Cleveland, OH 44106, USA

**Keywords:** Vaccine response, Machine learning, Antibody–virus interactions, Haemagglutination inhibition, Influenza, Prediction

## Abstract

**Background:**

Despite decades of research on the influenza virus, we still lack a predictive understanding of how vaccination reshapes each person’s antibody response, which impedes efforts to design better vaccines. Models using pre-vaccination antibody haemagglutination inhibition (HAI) titres against the vaccine strain alone poorly predict post-vaccination responses.

**Methods:**

We combined fifteen prior H3N2 influenza vaccine studies from 1997 to 2021, collectively containing 20,000 data points, and develop of a machine learning model that uses pre-vaccination HAI titres against multiple influenza variants to predict post-vaccination responses. To further test the model, four new vaccine studies were conducted in 2022–2023 spanning two geographic locations and three influenza vaccine types.

**Findings:**

The most predictive pre-vaccination features were HAI titres against the vaccine strain and against historical influenza variants, with smaller predictive power derived from age, sex, vaccine dose, and geographic location. The resulting model predicted future responses even when the vaccine strain or vaccine formulation changed. A pre-vaccination feature—the time between peak HAI across recent variants—distinguished large versus small post-vaccination responses with 73% accuracy. Model predictions against prior vaccine studies had 2.4-fold error (95% CI: 2.34–2.40x, no large outliers with >4-fold error), yielding more accurate and robust predictions than a null model with 3.2-fold error (95% CI: 3.12–3.21x, 12% large outliers). The four new vaccine studies presented here were predicted with comparable accuracy to the intrinsic 2-fold error of the experimental assay.

**Interpretation:**

A person’s pre-vaccination influenza HAI titres using multiple variants are highly predictive of their post-vaccination response. Many individuals exhibited little-to-no vaccine response, as exhibited by the null model’s accuracy, yet the machine learning model identified and accurately predicted both weak and strong responses with statistical superiority. Taken together, this approach paves the way to better utilise current influenza vaccines, especially for individuals that exhibit the weakest responses.

**Funding:**

10.13039/100000060NIAID, UCSD PREPARE Institute, LJI & Kyowa Kirin, Inc. (KKNA–Kyowa Kirin North America), UGA, 10.13039/100007311Cleveland Clinic, the 10.13039/100008065Georgia Research Alliance, and the Bodman family.

**Research in context**

**Evidence before this study**

Multiple studies have noted the heterogeneity in antibody responses following influenza vaccination. While this variability has been linked to factors including age, sex, and the pre-vaccination antibody response, we lack methods that can use such features to accurately predict individual responses before vaccination, limiting their practical utility. Most prior models have been tested in a single influenza season, yet a robust predictive framework must generalize to future seasons.

**Added value of this study**

We develop a machine learning algorithm trained on historic influenza H3N2 studies to predict individual responses in future seasons. The most predictive features were pre-vaccination antibody titres against both the vaccine strain and historical influenza variants. The model achieved 2.4-fold prediction accuracy, outperforming a null model (3.2-fold accuracy) across past datasets and four new vaccine studies. Notably, the model does not require explicit encoding of the vaccine strain but instead learns from prior responses to that strain.

**Implications of all the available evidence**

Predicting vaccine responses could help identify individuals unlikely to benefit from standard vaccines, guiding personalized vaccine strategies. Surprisingly, neither high-dose vaccines, different inactivated formulations, age, sex, nor geographic location significantly altered response patterns. The null model’s performance suggests that, while influenza vaccines elicit a measurable population-level antibody response, many individuals exhibit little-to-no response.

## Introduction

Although the number of influenza vaccine platforms has rapidly grown over the past years, we lack methods to predict which individuals will poorly respond to a vaccine or what modifications would improve outcomes. Given influenza’s rapid evolution, it is unclear which virus strain (or variant, both terms used interchangeably) will arise next. Currently, vaccine design is “virus-centric,” where the primary goal is to ensure the vaccine strain matches the dominant circulating strain in the coming season.^1^, ^2^, ^3^ To quantify the vaccine’s immunogenicity, these efforts are complemented by ferret and human studies, each with their owns strengths and shortcomings.^3^, ^4^, ^5^

Human studies examine how responses to an existing vaccine inhibit future variants, but they do not test vaccines composed of new candidate strains. Ferret studies can directly compare infection responses from candidate strains, yet these ferret data do not capture the heterogeneity nor the complex immune history in humans. Due to such limitations, influenza vaccine effectiveness remains around 20–50% even in seasons when the vaccine strain matches the dominant circulating strain, with a sizeable fraction of individuals showing no measurable vaccine response.^6^, ^7^, ^8^, ^9^ This underscores the need for a combined virus-and-people-centric approach based upon both a strain’s prevalence and the immunity it elicits in people.

Vaccination induces a complex cascade of immune interactions spanning innate and adaptive immunity.^5^ In this work, we focus on the antibody response that plays a central role in mediating protection against influenza, specifically focusing on the haemagglutination inhibition (HAI) assay that correlates with protection.^10^, ^11^, ^12^ HAI quantifies how antibodies within sera block the virus from binding sialic acids on red blood cells, with higher HAI titres associated with greater protection.^13^

**Fig. 1:**
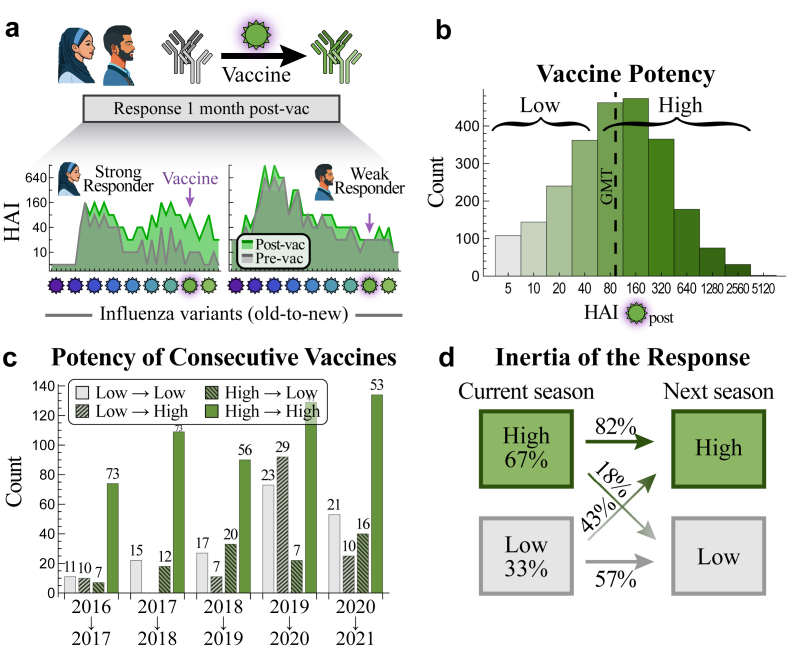
**Characteristics of the post-vaccination antibody response across multiple studies.** (a) Antibody inhibition against multiple variants is heterogeneous across the population. Two representative examples of a strong and weak vaccine response (IDs 69 and 117 from the 2016 Fox_HCW_ dataset, Table 1) showing pre-vac [grey] and 1-month post-vac HAIs [green]. The vaccine strain is denoted by a pink halo and variants are ordered from oldest-to-newest [purple-to-green viruses; past variants to the left of vaccine strain, future variants to its right]. (b) Distribution of vaccine strain HAI titres 1-month post-vac across the 15 prior studies from Table 1. HAI geometric mean titre (GMT = 95) is shown by a dashed line. (c) Tendency of post-vaccination titres to be high (HAI ≥ 80) or low (HAI ≤ 40) over two consecutive seasons (*x*-axis, all from the UGA studies). The percent in each class is shown above the bars. (d) Summary of high or low titres across all years (boxes) and the tendency to maintain these profiles over consecutive seasons (arrows).

Although numerous studies use HAI to assess vaccine responses, we lack frameworks that can predict each person’s vaccine response *a priori* and identify why the same vaccine elicits large HAI titres in some but low titres in others (Fig. 1a). Prior exposures may boost subsequent vaccine responses^14^^,^^15^ or prevent development of *de novo* B cell responses.^16^^,^^17^ Antigenic seniority, imprinting, vaccine blunting, and antibody ceiling effects have been observed, often at the population level, yet it is unclear how they weave together to shape each person’s post-vaccination response.^6^^,^^18^, ^19^, ^20^, ^21^

Indeed, using pre-vaccination (pre-vac) HAI to predict influenza responses in future seasons has proved challenging. Some studies found that vaccine responses in one season can predict others’ responses *in that same season*.^22^, ^23^, ^24^ Yet Parvandeh et al. showed that a model trained in one season (*R*^2^ = 0.63 training) can poorly generalize to *a different season* (*R*^2^ = 0.26–0.36 testing),^25^ with similar results found by Wu et al. (*R*^2^ = 0.25–0.38).^26^

Different modelling approaches have shown that certain aspects of the vaccine response are predictable. Groups opting for the simpler task of classification (whether responses meet a threshold such as HAI ≥ 40 or fold-change ≥ 4x), rather than directly predicting post-vaccination HAI, report accuracy as high as 89%.^22^, ^23^, ^24^^,^^27^, ^28^, ^29^ Others used data from the early vaccine response (days 1–7 post-vaccination) to further improve predictions.^27^ Despite these advances, we lack frameworks that can be used at the start of a season to pick the best vaccine candidate and forecast its post-vaccination HAI titres.

This work develops such a model that predicts exact post-vaccination HAI titres and tests it across influenza seasons from 2009 to 2021, with training always restricted to data from prior seasons. A key innovation from previous methods is that this model not only uses pre-vac HAI against the vaccine strain, but against dozens of historical variants, providing a shared feature space to combine past studies. Whereas models focusing exclusively on the vaccine strain deal with a moving target, our approach uses a set of variants that can be measured year after year to provide a fixed basis.

Variants also describe the antibody response with higher resolution. For example, they quantify how well the vaccine response cross-reacts against strains not included in the vaccine, a crucial metric given influenza’s rapid evolution and the potential for vaccine mismatch. Other groups have used variants to infer prior exposure history (e.g., did a substantial pre-vac HAI arise from a mild infection this year or a serious infection in a prior year^30^) and quantify imprinting effects, both of which shape future responses.^4^^,^^31^ Variants also provide a way to buffer the inherent error of the HAI assay, since a 4-fold increase in HAI titre against the vaccine strain is more credible when this same fold-change is seen against recent variants.

In addition to pre-vac HAI against the vaccine strain and variants, other host features (e.g., age,^18^^,^^32^^,^^33^ demographics,^26^ genomics^34^^,^^35^) have been shown to affect the antibody response, yet their effects are often assessed one study at a time, and it is unclear how much they improve vaccine predictions in future seasons. Here, we demonstrate how combining multiple studies readily quantifies how any combination of parameters constrains the post-vaccination response. The most predictive variables not only inform future study design but also provide the ingredients to model the vaccine response.

Using those maximally predictive variables, we develop an algorithm that takes a person’s HAI titres pre-vac to predict their peak HAI response 3–4 weeks post-vaccination (henceforth referred to as “1-month post-vac”). This analysis focuses exclusively on H3N2, since over half the datasets examined only measured this subtype. Our key findings include: (1) By combining data from prior vaccine studies, heterogeneous vaccine responses across ≥10 influenza seasons can be predicted with accuracy comparable to experimental noise. (2) Prediction accuracy holds across four new vaccine studies we conduct (in 2022 and 2023) spanning three vaccine types and two geographic locations. For that challenge, the computational team (T.E.) was blinded and only given the pre-vac data to stringently test the model’s predictive power. (3) The most informative variables for the post-vac response are an individual’s pre-vac HAI against the vaccine strain, their pre-vac HAI against other influenza variants, whether they receive an inactivated versus live attenuated vaccine, and their prior influenza exposure history. In comparison to these variables, age and other demographic information only marginally improved prediction accuracy. (4) While individual studies may find different relationships between variants, combining multiple studies leads to universal relations that accurately predict post-vac titres. (5) The magnitude of the fold-change post-vac is strongly associated with the number of years (Δ_Peak_) between the two most recent peaks in the HAI landscape; 2 ≤ Δ_Peak_ ≤ 3 yields a large fold-change while 4 ≤ Δ_Peak_ ≤ 6 leads to a smaller fold-change in 73% of cases.

The resulting algorithm is built upon a machine learning approach that finds relations between pre- and post-vac HAI titres from large-scale influenza vaccine studies going back to 1997. Instead of splitting each dataset into training/testing sets, we restrict ourselves to the harder prediction challenge of training on some datasets and testing on *entirely different datasets*. These zero-shot predictions are done forward in time; for example, to predict a person’s vaccine response in 2020, training was done on data from 2019 and before. Thus, each dataset provides external validation for this method, with the model going back in time to the start of each season and only training on prior data. Every variant measured in at least one prior study is predicted based on its pre-vaccination titres, without needing to “peek” at the initial vaccine response.^4^^,^^36^, ^37^, ^38^, ^39^, ^40^

## Methods

### Datasets analysed

We conducted a PubMed search (keywords: “influenza human vaccination H3N2 haemagglutination inhibition variants”) and manually searched for studies, or references within those studies, measuring vaccine responses in ≥25 people, ≥6 H3N2 variants, and where the data was either publicly available or could be requested from the authors. The resulting vaccine studies are described in the following manuscripts: Fonville,^4^ Hinojosa,^41^ Fox,^42^ UGA,^43^ Crotty [this work]. Vaccine strains are listed in Supplementary Fig. S1; in 14/16 studies the vaccine strain was included in the variant panel, but in two cases the closest variant was used instead (2009 Fonville: A/Perth/27/2007 [ΔAA_Epitope_ = 1 substitution in the HA head compared to A/Brisbane/10/2007], 2021 UGA: A/Tasmania/503/2020 [ΔAA_Epitope_ = 2 substitutions compared to A/Cambodia/e0826360/2020]). Each study’s name contains the year of vaccination (e.g., 2016 Fox_Nam_ represents a 2016 vaccine study).

The explicit time points measured in each study can subtly differ. 2016 Fox_Nam/HCW_ and 2016/2017 UGA measured the response 21 days post-vac, whereas all other vaccine studies measured the day 28 post-vac response. In 2020/2021 UGA, measurements were given as a spectrum of more precise dates (i.e., participants were asked to return 28 days post-vaccination, but the exact date of the post-vac visit was recorded. This exact date ranged from 21 to 71 days, and a similar spread is expected in all other studies). Such subtleties in timing were ignored in this analysis, with all post-vaccination time points treated as “1-month post-vaccination,” which simplifies time from a continuous variable to a Boolean variable.

Sex was treated as a biological variable. In our new vaccine studies, all participants were allowed to enrol regardless of their sex/gender, and the resulting cohorts were balanced across sex (Supplementary Fig. S1).

### Variants

The full list of H3N2 variants measured in each dataset are given in Supplementary Fig. S2c. The four new vaccine studies conducted in this work measured HAI titres against A/Hong Kong/4801/2014 (except 2023 UGA), A/Singapore/INFIMH-160019/2016, A/Kansas/14/2017, A/South Australia/34/2019, A/Hong Kong/2671/2019, A/Tasmania/503/2020, and A/Darwin/9/2021. Most viruses were propagated in embryonated chicken eggs in the lab of Dr. Ted Ross for all four new vaccine studies. The only exceptions were A/Darwin/9/2021 and A/Kansas/14/2017 in the 2023 Crotty_Afluria/FluMist_ studies, which due to limited reagents were instead supplied by Dr. Florian Krammer.

All vaccine strains were egg-grown, and all variants from the Hinojosa, UGA, and Crotty studies were egg-propagated. Variants from the Fox studies were mostly cell-passaged (see Table 1 from that reference^42^), while the Fonville study did not report passaging information. However, there was little difference between the HAIs of egg- versus cell-passaged variants (Supplementary Fig. S2e), and such differences were ignored in the model.

Analogous variants differing by ΔAA_Epitope_ ≤ 5 were equated in the order of their smallest ΔAA_Epitope_ values (Supplementary Fig. S2b). In case of ties, virus analogues that increased the overlap across all studies were prioritised. 3-way analogues (e.g., A/Hong Kong/1/1968 ≈ A/Bilthoven/16190/1968 ≈ A/Aichi/2/1968) were only considered if ΔAA_Epitope_ ≤ 5 across all pairs.

### Vaccine study participants

25 participants were recruited for each of the four new influenza vaccine studies presented in this work (2022 UGA, 2023 UGA, 2023 Crotty_Afluria_, and 2023 Crotty_FluMist_). These studies administered different formulations of the influenza vaccine from that season.

The 2022 UGA study administered the 2022–23 vaccine comprising H1N1 A/Victoria/2570/2019, H3N2 A/Darwin/9/2021, B/Austria/1359417/2021 (B/Victoria lineage), and B/Phuket/3073/2013 (B/Yamagata lineage). As in prior UGA studies, participants less than 65 years old were given standard-dose [15 mg/component] Fluzone Quadrivalent (Sanofi Pasteur). Participants aged 65 or older were offered the high-dose [60 mg/component] Fluzone Quadrivalent (Sanofi Pasteur), and 3/5 of participants in this age group opted for the high-dose vaccine (see GitHub data for information on vaccine dose).

All three 2023 studies administered the 2023–24 vaccine composed of H1N1 A/Victoria/4897/2022 and the same H3N2, B Victoria, and B Yamagata strains as the 2022–23 vaccine. In the 2023 UGA study, participants under 65 years old were given standard-dose Fluzone Quadrivalent (Sanofi Pasteur) while those 65 and older were offered the high-dose version, with 9/10 participants in this age group opting for the high-dose vaccine. All participants in the 2023 Crotty_Afluria_ study received the 2023–24 formulation of Afluria Quadrivalent (Seqirus) while those in 2023 Crotty_FluMist_ received FluMist Quadrivalent (AstraZeneca).

In all studies, sera were collected pre-vaccination (day 0) and one-month post-vaccination (day 28) in the fall of their respective year. Participants for the 2022 UGA and 2023 UGA studies were recruited from medical facilities near Athens GA while those in the Crotty studies were recruited from La Jolla CA. The SST tube of one participant in 2023 Crotty_Afluria_ clotted during the one-month blood draw resulting in *n* = 24 post-vaccination samples, and the unpaired pre-vaccination sample was dropped from our analysis.

### Analysing HAI titres

The haemagglutination inhibition (HAI) assay quantifies how potently serum inhibits a virus from binding red blood cells, with larger titres representing a more potent serum. This assay is done using 2-fold dilutions, so HAI titres can equal 10, 20, 40… Plots with too many overlapping points were jittered for clarity. Sera that did not inhibit haemagglutination at the lowest dilution (HAI <10) were denoted by HAI = 5.

As in previous studies, all analysis was done on log_10_(HAI titres) because experimental measurements span orders of magnitude, and taking the logarithm prevents biasing the predictions toward the largest values while also accounting for the declining marginal protection from increasing titres.^10^

### Intrinsic noise of the HAI assay

Prior work has shown that the HAI assay has roughly 2x error. Harvey et al. reported HAI from influenza surveillance data, where HAI from the same ferret sera were measured against the most common circulating variants each week.^44^ Their dataset contained ∼2,300,000 repeat measurements of the same serum–virus pair, and these measurements were consistent with Gaussian error (on a log_2_ scale) with standard deviation *σ* = 1 (i.e., 2-fold error). More precisely, 40.0% of their repeat measurements did not change (1x error), 42.5% had 2x error, 12.6% had 4x error, 3.5% had 8x, and 1.0% had 16x error. In comparison, a log-Gaussian error distribution predicts that 38.7% of measurements would not change, 48.3% have 2x error, 11.7% have 4x error, 1.1% have 8x error, and 0.03% have 16x error.

Fonville et al. analysed HAI measurements from nearly identical sera and found that the inherent error of the assay is log-normally-distributed with standard deviation ≈2-fold.^4^ This is shown by Fig. S8B in Fonville et al.^4^ (neglecting the stack of not-determined measurements outside the dynamic range of the assay), where 40% of repeats had the same HAI value, 50% had a 2-fold discrepancy, and 10% had a 4-fold discrepancy. Note that because HAI < 10 is reported as HAI = 5, the few variants with very low HAI (such as Hong Kong 1968 and Port Chalmers 1973 in 2017 UGA) may artificially achieve an error <2x, since an HAI = 2.5 will be reported as HAI = 5 even with 2x error. However, most variants have appreciable titres and exhibit the expected 2x error.

Across the 15 studies examined in this work, there were *n* = 9 pairs of variants with identical HA sequences that were measured within the same study. The RMSE between the HAI of these 9 variants varied from 1.6x–3.3x with a geometric mean of 2.1x. Taken together, these results demonstrate that the HAI assay has 2x error, and hence HAI prediction that achieve an RMSE ≈2x are as accurate as possible given experimental noise.

### HAI protocol

For the UGA studies, sera were treated with receptor destroying enzyme (RDE) (Denka) to inactivate nonspecific inhibitors. Briefly, three volumes of RDE were added to one volume of sera and incubated overnight at 37°C. The next day, samples were incubated at 56°C for 30–60 min, after which 6 volumes of 1x phosphate buffered saline (PBS) were added to each sample, resulting in a final serum dilution of 1:10. For the Crotty studies, 20 μL starting volume of serum was treated with 0.5 × (starting volume) of 8 mg/mL TPCK-trypsin (Sigma-Aldrich) and incubated at 56°C for 30 min. After cooling to room temperature, 3 × (starting volume) of 11 mM potassium periodate (Sigma-Aldrich) was added and incubated for 15 min at room temperature. 3 × (starting volume) of 1% glycerol-PBS was then added and incubated for 15 min. Lastly, 2.5 × (starting volume) of 0.85% PBS was added to all samples.

Sera were diluted in a series of 2-fold serial dilutions in 96-well V-bottom plates (Thermo Fisher for UGA studies, Sarstedt for Crotty studies). An equal volume of influenza virus, adjusted to 8 haemagglutination units (HAU)/50 μL diluted in 1x PBS, was added to each well of the plate. For UGA studies, plates were then covered and allowed to incubate at room temperature for 20 min. After incubation, 50 μL of a solution consisting of 0.8% turkey red blood cells (Lampire Biologicals) diluted in 1x PBS was added to each well. The plates were then mixed by gentle agitation, covered, and allowed to incubate for another 30 min at room temperature. For Crotty studies, sera and virus were incubated for 30 min at room temperature. After this incubation, 50 μL of 0.5% turkey red blood cells (Lampire Biologicals) or 0.75% guinea pig red blood cells (GPRBCs) in 1x PBS (Lampire Biologicals) were added to all wells and incubated at 4°C for 45 min. GPRBCs were used for H3N2 A/Darwin/9/2021 in the Crotty studies due to lack of viral activity observed with turkey red blood cells.

After incubation with red blood cells, plates were tilted to observe haemagglutination inhibition. The HAI antibody titre was determined by taking the reciprocal dilution of the last well that contained non-agglutinated red blood cells. For GPRBC plates, the HAI titre was determined as the last dilution with a “halo” of the same size as PBS and virus only wells. Samples with no detectable activity were assigned to half the limit of detection (HAI = 5).

To confirm assay consistency between runs, positive controls were included. In the UGA studies, these consisted of sera from previously performed mouse or ferret infections. In the Crotty studies, normal control goat serum (FR-1377) and positive control influenza A (H3) Reference Goat Antiserum (FR-1562, FR-1612, FR-1683, FR-1737, FR-1780, FR-1827) were obtained through the International Reagent Resource, Influenza Division, WHO Collaborating Center for Surveillance, Epidemiology and Control of Influenza, Centres for Disease Control and Prevention, Atlanta, GA, USA. In addition, pre- and 1-month post-vaccination serum for the same participants were run on the same plate with negative and positive control serum.

### Quantifying predictive power of different features

When assessing feature importance, a pair of subjects were said to be matched across the following features provided they satisfied:

• *Age*: Pairs have ages ≤10 years apart from one another. (Alternative thresholds of ≤5 and ≤20 years apart led to worse predictions.)
• *Sex*: Pairs have the same sex (male or female).
• *Vaccine dose*: Pairs receive the same standard-dose or high-dose vaccine. (The latter is only available, but not mandatory, when age ≥65.)
• *Location*: Pairs exactly matched along the geographic location of the study (Fonville studies or 2016 Fox_HCW_→Australia, 2016 Fox_Nam_→Ha Nam, Hinojosa studies→Wisconsin, UGA studies→Georgia).
• *Pre-vac HAI* (Vac_Pre_): Pairs exactly match in their pre-vaccination HAI titre against the respective vaccine strain in their study.
• *Variant HAI*: Pairs must be measured against ≥5 overlapping viruses (excluding their vaccine strains), and there must be a correlation >0.9 between the log_10_(HAI titres) of these overlapping viruses.

If no features were used, then all pairs of subjects were considered to establish a baseline prediction accuracy. Prior vaccination history was not known in the Fonville or Hinojosa datasets and was not used in the modelling. The ratio of post-vac titres was always restricted to be ≥1.

We also explored additional ways to encode pre-vac HAI against variants to determine whether individuals matching with this transformed feature had more similar post-vac HAI. We considered area under the curve (*x* = virus year, *y* = HAI) or taking the geometric mean HAI across all variants, where in both cases we either used all variants or a single variant from each antigenic cluster in Fonville et al.^4^ We also assessed a 3D analogue, using the volume of the convex hull where each variant’s (*x*, *y*) coordinates are given by antigenic cartography and its *z*-coordinate is given by its HAI. Each transformation led to post-vac RMSE ≳4x, demonstrating less predictive power than directly using the vector of pre-vac HAI against the vaccine strain and variants.

### Predicting the HAI response 1-month post-vaccination

The random forest model builds upon prior work,^39^ with each decision tree constrained to use pre-vaccination data as input and post-vaccination data as output. Briefly, to predict a virus *V*_0_ from Dataset_1_→Dataset_2_, we chose five overlapping variants (*V*_1_–*V*_5_, with replacement, at least one must be *V*_0_) and trained a decision tree that uses day 0 HAI from *V*_1_–*V*_5_ to predict day 28 HAI from *V*_0_. Each decision tree was trained on a random 30% of the sera in Dataset_1_, and then cross-validated on the remaining 70% of sera. This process was repeated 50 times for each *V*_0_, and the five best decision trees with the lowest cross-validation error were combined into a small random forest that was then applied to Dataset_2_ to predict *V*_0_. Decision trees were created using the *Predict* function with the “DecisionTree” Method and default hyperparameters in *Mathematica* (version 14.1).

We required two datasets to measure ≥4 overlapping variants from which *V*_1_–*V*_5_ were drawn. Since predictions were done forward in time (e.g., we only predict from Dataset_1_→Dataset_2_ if Dataset_2_ was conducted at least one year after Dataset_1_), predictions for any variant *V*_0_ in Dataset_2_ are only possible if *V*_0_ was measured in a prior study. As an example, the new 2018 vaccine strain H3N2 A/Singapore/INFIMH-16-0019/2016 could be predicted in 2018 UGA because it was first measured in 2017 UGA. Similarly, the vaccine strains in 10/15 of the studies in Table 1 could be predicted because those vaccine strains had been previously measured.

**Table 1:** **Large scale influenza vaccinestudies analysed in this work.**

Fifteen existing vaccine studies (white background) were used to train and test the model forward in time. Four new vaccine studies (grey) were exclusively used to test the model. The total number of measurements in each study equals (# of sera) × (# of viruses) × (2 time points [pre- and post-vac]).

^a^Each year represents the date when a vaccine study was conducted, not when the corresponding manuscript was published. ^b^All vaccine strains were egg-grown, as were most test strains (Methods). High dose vaccines were only offered to individuals age ≥65 in the 2016–2023 UGA studies.

Since the pre-vac HAI titres vary across a cohort, the specific choice for the 30% of sera used to train a decision tree predicting *V*_0_ in Dataset_1_ matter. Thus, we explored three types of decision trees to potentially offset such bias. The first type randomly sampled sera in Dataset_1_ irrespective of their pre-vac HAIs. The second evenly sampled across bins of *V*_0_‘s pre-vac HAI (with bins [5, 10, 20 … 640, and ≥1280]) to prevent training data dominated by low HAI titres. Third, we added five “null model” decision trees that only uses *V*_0_’s pre-vac HAI to predicts its post-vac HAI (i.e., *V*_0_=*V*_1_=*V*_2_=*V*_3_=*V*_4_=*V*_5_), randomly choosing the training sera with a uniform distribution. This final group of decision trees is necessary for the oldest variants that show little-to-no vaccine response. All three types of decision trees were combined and collectively assessed based on their cross-validation RMSE on the left-out subjects in Dataset_1_, and the average of the five trees with the lowest cross-validation RMSE were used to predict cross-study responses.

Batch effects across and within studies were handled by row-centring data, which we found markedly improved prediction accuracy. Row-centring means that if *t*_0_–*t*_5_ represent the log_10_ (titres) of *V*_0_–*V*_5_, with mean titre *t*_avg_, then each decision tree will take (*t*_1_-*t*_avg_, *t*_2_-*t*_avg_, *t*_3_-*t*_avg_, *t*_4_-*t*_avg_, *t*_5_-*t*_avg_) as input to predict *t*_0_-*t*_avg_. The value *t*_avg_ (which will be different for each serum) is then added to this prediction to undo the row-centring. As an example, if all data in one study was systematically increased by 2x, row centring ensures that predictions in other datasets would not change, and that predictions within the shifted study would increase by 2x. Note that row-centring does not throw away any information, nor does it normalize or threshold the HAI titres. Instead, the exact post-vaccination titres were predicted for each variant.

When using multiple training sets, predictions from each study were equally weighed by taking their geometric mean (or the arithmetic mean of the log_10_[HAI titres]). Unequal weights (for predictions from 1, 2, 3… prior seasons) were assessed through nonlinear fitting, yet the optimal weights found were essentially flat, and hence equal weighing was used.

Hyperparameters were adopted from prior work,^39^ but also tested across a range of values to minimize RMSE and maximize the coefficient of determination *R*^2^ across the vaccine strains. For example, when predicting H3N2 A/Hong Kong/4801/2014 in the 2017 UGA study, we tested random forests that used 2 (RMSE = 3.3, *R*^2^ = −0.4), 3 (RMSE = 3.0, *R*^2^ = −0.1), 4 (RMSE = 2.4, *R*^2^ = 0.3), or 5 (RMSE = 2.4, *R*^2^ = 0.3) variants as input, choosing to use 5 variants. We further tested whether the random forest should be comprised of 3, 5, 7, or 9 top trees and, finding little variability (RMSE = 2.4, *R*^2^ = 0.2–0.3), opted for 5 trees in each forest.

The final post-vac predictions were given as the maximum of the pre-vac HAI and the model predictions. Although there were very few cases where model predictions fell below the pre-vac titres, this biologically-realistic constraint slightly improved model predictions.

Null and linear models were used to provide a baseline for prediction accuracy. The null or “no response” model assumed that each person’s post-vac HAI titre equals their pre-vac titre for each variant. The linear model assumed that log_10_HAI_post_ = *c*_1_ + *c*_2_·log_10_HAI_pre_, with different constants *c*_1_ and *c*_2_ inferred for each variant in each study based on the average of the coefficients determined from all prior studies.

### Classifying robustly strong and robustly weak responses

In addition to the random forest (regression) model, two classification models were trained using either nearest neighbours or a support vector machine (SVM), with the SVM using the sigmoid kernel *K*(*x*_1_,*x*_2_) = tanh(*c* + *γ x*_1_·*x*_2_). Each instance of the nearest neighbour and SVM were separately trained on every dataset, taking in all subjects’ pre-vac HAI against all variants and a classification of “strong” if post-vac GMT/pre-vac GMT ≥2.5 (which requires a substantial post-vac response, especially since HAI from older variants stays nearly constant post-vac) and “weak” if post-vac GMT/pre-vac GMT ≤ 1.1 (a null response, with the threshold slightly above 1.0 to account for intrinsic error in the HAI assay). Subjects with an intermediate fold-change were not considered. The resulting models were applied to each subject’s pre-vac HAIs as well as every perturbed version of their HAIs (where each titre was increased or decreased by 2x), and cases where ≥75% of states were strong/weak were denoted as robustly strong/weak.

We determined the “peaks” in HAI data by first sorting viruses by their year of circulation. Only northern hemisphere vaccine strains were considered to ensure there was at most 1 variant per year (since otherwise the order of those variants would be ambiguous); in practice this only removed the 2019 southern hemisphere strain, H3N2 A/South Australia/34/2019. Peaks were then determined after applying a Gaussian blur with scale *σ* = 1 to account for error. The depth of each peak (i.e., whether HAI decreases by 2x or more) was not considered. In case of a plateau where multiple consecutive variants had an equally large HAI, the peak was set at the position of the earliest variant.

The two most recent peaks between (year of study)‒6 and (year of study) were used to compute Δ_Peak_; if two peaks did not exist in this time frame then no classification was made. Attempts to use values of Δ_Peak_ > 6 to classify lead to worse accuracy, likely because the variant panels lose resolution beyond this time interval and hence many peaks are artificially missed.

### Statistics

Prediction error was quantified in unlogged units so that it can be readily compared to the measured values. RMSEs were computed by first taking the root-mean-squared error *σ* of the log_10_(HAI titres) and then exponentiated by 10 (i.e., *σ* = 0.3 for log_10_ titres corresponds to an error of *σ*_Predict_=10^0.3^=2-fold, with “fold” or “x” indicating an un-logged number). Confidence intervals were computed by bootstrapping the log_10_ values.

As an example, if a feature set would yield *n* = 5 participants whose post-vac HAI against the vaccine strain differs by (1x, 1x, 2x, 2x, 4x), their RMSE would be 2.14x and their confidence interval is defined as the span between the 5th and 95th elements in the list created as follows: choose *n* = 5 entries from this list with replacement, calculate their RMSE, and repeat this process 100 times. For the example above, the 95% CI would be 1.36–2.93x.

### Ethics

The UGA vaccine studies were approved by the Western Institutional Review Board and the University of Georgia Review Board (IRB #20224877). The Crotty studies were approved by the La Jolla Institute for Immunology Review Board (IRB #VD-271). All participants provided informed consent.

### Role of funding sources

Funding sources played no role in the writing, analysis, or submission of this manuscript. The authors were not precluded from accessing study data, and they all agreed to and accepted responsibility to submit the manuscript.

## Results

### Curating influenza vaccine studies to compare responses across datasets

We performed an extensive literature search for influenza vaccine studies from the past two decades that measured responses against multiple variants. Due to the substantial effort involved, such studies often restricted their analyses to the sizable datasets they produced. Here, we chose the opposite tact and characterised responses across cohorts. Doing so builds towards a fundamentally new question, namely, whether all post-vaccination HAI data in each study can be entirely predicted by prior studies, even as the virus evolves and the vaccine strains change.

The literature search found 15 influenza vaccine studies measuring pre- and post-vac serum HAI in ≥25 people against ≥6 H3N2 variants (Table 1, Supplementary Fig. S1). On average, cohorts included 160 (from 31 to 461) sera that showcase the heterogeneity of responses, measured against 30 H3N2 variants (from 7 to 70). Collectively, these ∼2500 sera measure vaccine responses from much of the past decade (2014–2021) as well as four prior years (1997, 1998, 2009, 2010), providing ample opportunities to characterise vaccine responses within and across influenza seasons. HAI titres are row-centred to account for batch effects across studies (Methods).^39^

### The inertia of vaccine responses elicits consistently high/low titres across seasons, especially when the vaccine strain is unchanged

We first examined the inertia of vaccine responses, namely, how conserved a person’s response is across seasons. For this section only, we examined the variability in dichotomous outcomes (low versus high post-vac titre) before creating a framework that predicts the specific post-vac titre. The vaccine strain’s HAI titre 1-month post-vac followed an approximate log-normal distribution with a geometric mean titre of GMT = 95 (*n* = 2441, Fig. 1b). Classic studies found that an HAI titre of 40 or 80 correlated with 50% protection against H3N2 infections.^45^, ^46^, ^47^, ^48^ Adopting the conservative stance where HAI ≥80 represents a high titre that confers this protection, 67% of sera had high post-vac titres across studies (Only 37% of sera had high titres pre-vac, so roughly half of these cases are attributed to high preexisting inhibition and the other half were elicited by the vaccine.)

Individuals vaccinated in two consecutive seasons showed an inertial tendency towards maintaining their high or low titres, although there was a global bias towards high titres (Fig. 1c and d). More precisely, someone exhibiting a high titre was 82%/18%≈4x as likely to exhibit a high titre the next season, while someone with a low titre was only 57%/43% ≈1.3x as likely to maintain their low titre (Fig. 1d).

Across all years, the fraction of individuals that maintained their high→high or low→low titre was 73% (=0.82·67% + 0.57·33%, Fig. 1d), yet ≥83% did so during seasons when the H3N2 vaccine strain was unchanged or minimally changed (Supplementary Table S1, Spearman's rank test *p* = 0.04 using the HA epitopes [ΔAA_Epitope_], *p* = 0.02 using full HA [ΔAA_Total_]). Taken together, these results demonstrate that features of the vaccine strain and the antibody response from (at least) one prior season can help inform future responses.

### A model-free approach to quantifying predictive power of host traits

In addition to past vaccine responses, other factors affect the antibody response including age,^18^^,^^21^^,^^49^, ^50^, ^51^ genetics,^27^^,^^52^ and a person’s vaccination or infection history.^42^^,^^53^, ^54^, ^55^ Yet the amount each factor, or a combination of factors, affects the response is harder to quantify. Powered by these 15 studies, we introduced a simple, model-free method to assess how different combinations of features constrain the post-vac HAI against the vaccine strain. This approach predicted an individual’s HAI titre based on the titres of “matched” individuals from prior seasons that share a prespecified set of features.

As an example, we first assessed two commonly measured features: a person’s age and their pre-vac HAI against the vaccine strain. To quantify how these two features constrain the post-vac response, every pair of individuals that have a similar age and pre-vac HAI (age ≤ 10 years apart, HAI exactly matches) were considered, and the ratio of their vaccine strain’s HAI 1-month post-vac was computed (which should be ≈1 if they exhibit similar responses and >1 if they differ).

**Fig. 2:**
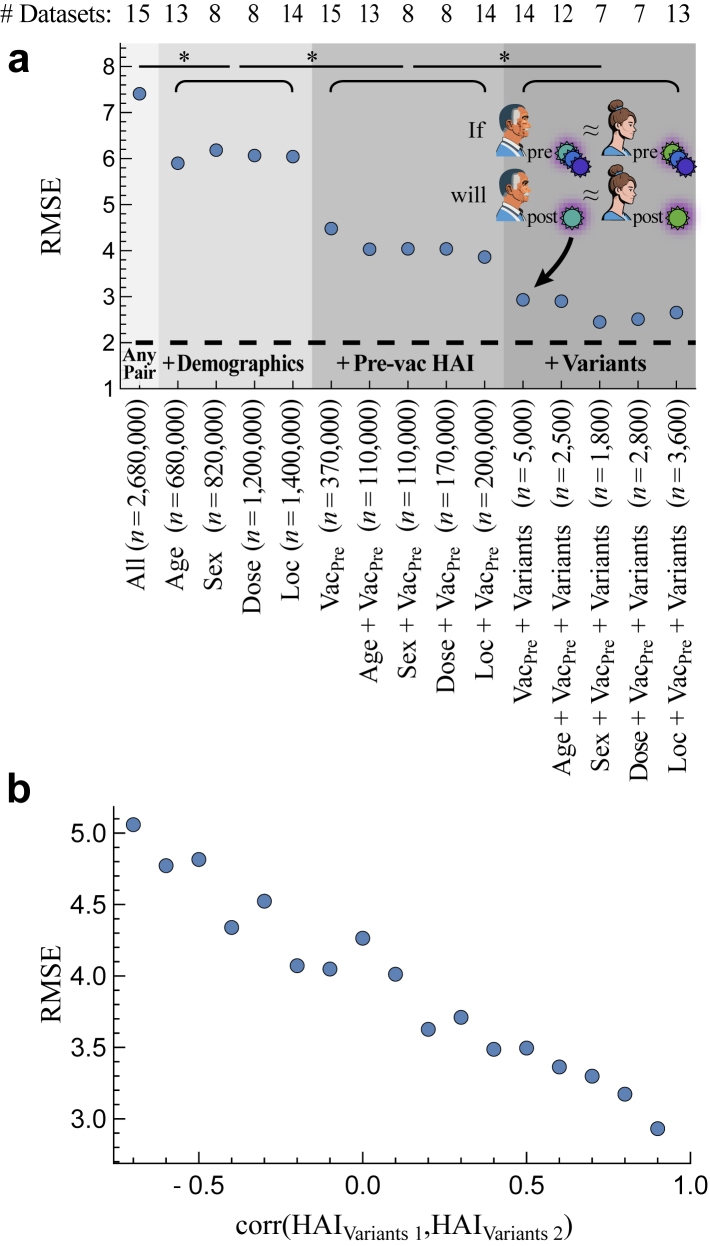
**A model-free way to identify the key parameters that predict vaccine responses across studies.** (a) For all pairs of subjects vaccinated in different seasons that match across a feature set [*x*-axis], their post-vac HAI was compared against their respective vaccine strains. Features include standard versus high dosage (Dose), geographic location (Loc), pre-vac HAI against the vaccine strain (Vac_Pre_), and pre-vac HAI against other variants (Variants); None means no feature were applied (Methods). Root-mean-squared error (RMSE, *y*-axis) is shown for each feature set, assessed over *n* pairs of subjects. 95% CI are smaller than the plot markers. The dashed line represents the intrinsic 2-fold error of the HAI assay.^4^ ∗*p* < 0.001 between any feature sets with different grey shading (e.g., Age versus Vac_Pre_ or Sex versus Vac_Pre_+Variants) using a one-sided permutation test between post-vac HAI fold-change across pairs in each column. (b) Using the individuals in the *n* = 5000 pairs matching in Vac_Pre_+Variants, the correlation of pre-vaccination HAI across all variants (*x*-axis; e.g., *x* = 0.5 represents pairs with 0.5 < correlation ≤ 0.6) is shown versus the difference in their post-vaccination HAI against the vaccine strain (RMSE across all pairs with the same *x*-coordinate).

Given the ultimate goal of predicting vaccine responses across seasons, only pairs of subjects vaccinated in *different* years were considered to test how well these features constrain post-vac HAI, with each person’s titres assessed against the vaccine strain from their respective season. As a baseline, the post-vac HAI across all pairs differed by 7.4x root-mean-squared error (RMSE, “x” denotes fold-error; 95% CI: 7.40–7.42x). For the specific case of age and pre-vac HAI, *n*≈110,000 pairs matched on both traits across all studies with RMSE = 4.0x (95% CI: 4.01–4.06x) (Fig. 2a, Supplementary Fig. S3a Age + Vac_Pre_). By assessing each feature separately, most of this predictive power came from the pre-vac HAI (RMSE = 4.5x, 95% CI: 4.46–4.49x, Fig. 2a Vac_Pre_). Age alone poorly constrained the post-vac response across all age groups (RMSE = 5.9x, 95% CI: 5.88–5.92x, Fig. 2a Age), although exclusively considering ages ≥65 led to more homogeneous responses and slightly better prediction accuracy (Supplementary Fig. S3b).

We similarly assessed other combinations of features measured in the 15 studies—including a participant’s age, sex, dosage, geographic location, pre-vac HAI against the vaccine strain, and pre-vac HAI against other variants—with matching specified by equality (e.g., sex_1_ = sex_2_), a tolerated range (|age_1_-age_2_|≤10), or correlation (corr [HAI_Variants 1_, HAI_Variants 2_]>0.9) depending on the variable (Methods). Among these features, the largest decrease in RMSE occurred when matching the pre-vac HAI against the vaccine strain (Fig. 2a Vac_Pre_), followed by matching pre-vac HAI across all variants (Fig. 2a Vac_Pre_ + Variants), both of which significant decreased RMSE (*p* < 0.001, one-sided permutation test, Fig. 2a).

To verify that the subset of *n* = 5000 pairs matching in Vac_Pre_ + Variants was not intrinsically more predictable than the Vac_Pre_-only group, the correlation across variants was computed between any two subjects in the former group. As expected, there was a strong and consistent trend where higher correlation led to more similar post-vac HAI (Fig. 2b). Since matching vaccine and variant HAIs led to more post-vac HAI ≥40 compared to other features that could bias results (Supplementary Fig. S3a Vac_Pre_ + Variants), we reevaluated prediction error after removing post-vac HAI titres <40 and found smaller but still significant decreases to RMSE from both the vaccine strain and variant HAIs (e.g., Age: 3.9x, Vac_Pre_: 3.5x, Age + Vac_Pre_: 3.3x, Vac_Pre_ + Variants: 2.8x).

Notably, adding any other features to pre-vac HAI led to smaller and less robust decreases in RMSE (Fig. 2a). These other features, ordered from most-to-least predictive power, were the geographic location, age, sex, and vaccine dose, each providing smaller but still significant decreases (*p* < 0.001 for all, one-sided permutation test, Fig. 2a). However, none of these features led to consistently improved accuracy in combination with other variables (age or sex shown in Supplementary Fig. S3b), suggesting that pre-vac HAI represents the most predictive variable in our analysis that controls post-vac HAI. Therefore, we proceeded with the parsimonious model using the pre-vac HAI against the vaccine strain and variants in the following sections.

### Forecasting post-vaccination HAI titres in future seasons

We next determined how well pre-vac HAIs predict post-vac titres, while taking into account the heterogeneity of responses, the different variants measured in each study, and differences in study design that may affect the response. Predictions were built upon a series of row-centred random forests, with a separate forest predicting each variant’s post-vac HAI (Methods). In total, ∼10^3^ separate models were collectively built to forecast the vaccine response.

Rather than splitting each dataset into a training and testing set—which for future application would require some individuals to get vaccinated early each season to predict others’ responses—we instead strive for the tougher challenge of training exclusively on vaccine studies from prior years. Each older study predicts every future study with sufficient overlap (≥4 overlapping variants, Supplementary Fig. S4), irrespective of their vaccine strains.

Since many influenza studies (beyond the ones analysed in this work) only measure the vaccine strain, we sought to beat the 4.5x error found when only matching the vaccine strain’s pre-vaccination HAI (Fig. 2A Vac_Pre_), ideally aiming for the 2x noise limit of the HAI assay (see Methods for the quantification of assay noise). We also compare model performance against a null model that assumed no HAI response (HAI_post_ = HAI_pre_) and a linear model for each variant (log_10_HAI_post_ = *c*_1_+*c*_2_·log_10_HAI_pre_, using logged titres for scale invariance).

### Equating analogous variants across studies

**Fig. 3:**
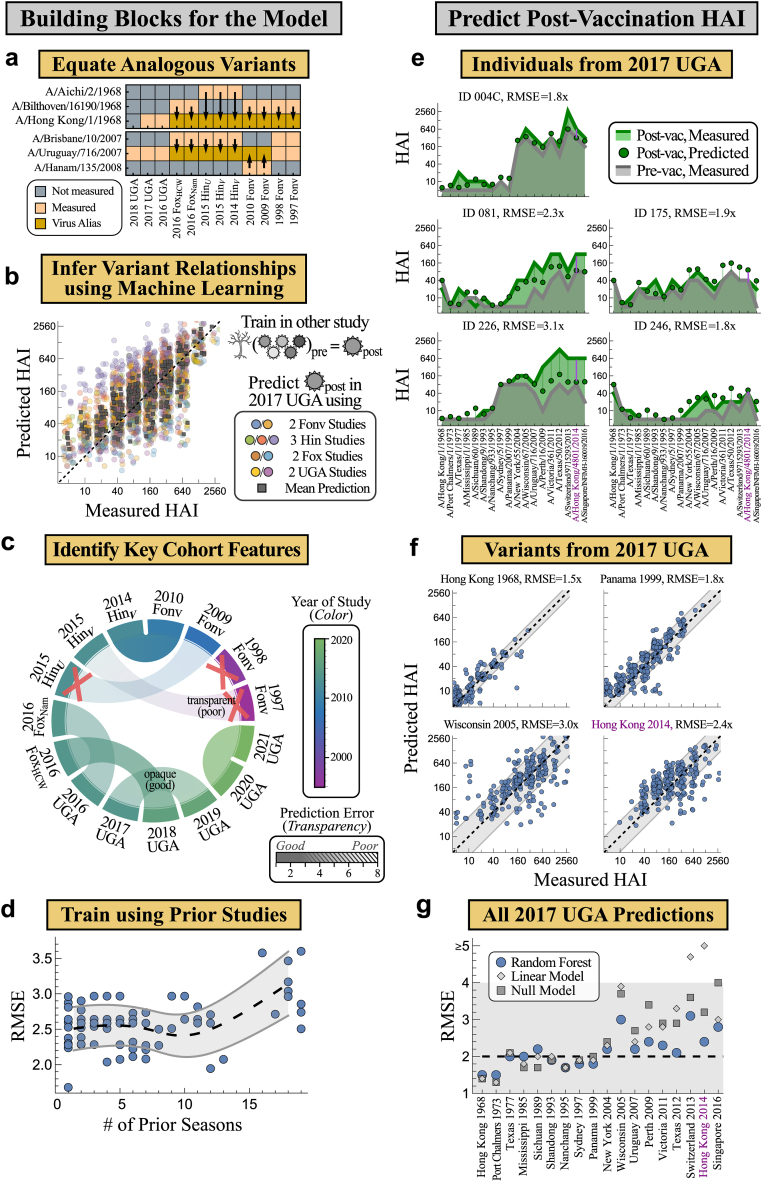
**Using pre-vaccination HAI against multiple variants to predict HAI 1-month post-vaccination.** (a) Analogous variants with similar HA sequences are equated (full list in Supplementary Fig. S2). Pre-vac HAI from these overlapping variants served as model input to predict post-vac HAI. (b) Example predictions for H3N2 A/Uruguay/716/2007 in 2017 UGA using 2009–2010 Fonville, all Hinojosa and Fox studies, 2016 UGA, and 2018 UGA (circles), together with the geometric mean across all 9 studies (squares). In every study, decision trees were trained using pre-vac HAI from five variants to predict Uruguay 2007’s post-vac HAI, and these trees were then applied to 2017 UGA. Each point represents one subject. (c) Representative chords showing prediction RMSE across pairs of studies, where more transparency corresponds to higher prediction error (full chords in Supplementary Fig. S6, legend shows a transparency gradient above a striped pattern). Colour represents the year a study was conducted but does not impact its transparency. The three crossed-out studies have upper quartile RMSE >4x. (d) Prediction error from training on all vaccine studies from the prior *n* seasons [*x*-axis]. Mean [black dashed] and standard deviation [grey lines] of these RMSEs are interpolated across *n*. (e) Example predictions of five individuals in 2017 UGA [subject IDs and RMSE across variants shown above]. Pre-vac HAIs [grey line] predicted the post-vac response [green points], and results were compared to post-vac data [green line]. Prediction error is emphasized by the vertical lines [purple bars and text highlight the vaccine strain H3N2 A/Hong Kong/4801/2014, also shown in the bottom-right of Panel f and in Panel g]. Variants are sorted from oldest to newest (left-to-right). (f) Predicted versus measured post-vac HAI of four variants across all 271 subjects in 2017 UGA. The diagonal line *y* = *x* represents perfect predictions, while the grey bands denote the RMSE of the predictions. (g) RMSE for all variants in 2017 UGA comparing the random forest approach (blue points) with a linear model (grey squares) or null model (grey diamonds). The grey region denotes predictions with the desired RMSE ≤4x.

Since predictions were based on the HAI of overlapping variants between studies, we increased this overlap by equating variants whose HA sequences differed by ΔAA_Epitope_ < 5 amino acids in H3N2 epitopes A-E. This threshold balanced the twin goals of equating more strains while ensuring that equated strains have similar HAI profiles (Supplementary Fig. S2a). For example, HAI from H3N2 A/Hong Kong/1/1968 ≈ A/Bilthoven/16190/1968 ≈ A/Aichi/2/1968 were combined for model training and testing (Fig. 3a; full list in Supplementary Fig. S2). Variants with multiple analogous strains were matched to minimize ΔAA_Epitope_ and maximize the overlap across studies (Methods). In total, this decreased the total number of unique variants across all studies from 102 to 80.

All subsequent analysis was carried out after equating these analogous strains. In all cases where virus analogues were measured in the same study, their HAI titres were nearly identical, as expected (Supplementary Fig. S2). However, prior work has shown that some single substitutions lead to dramatically different HAI reactivity,^56^ and hence the ultimate test for these equivalences will be the accuracy of the final forecasts.

### Inferring global relationships between the pre- and post-vaccination HAI of influenza variants

To predict the HAI for virus-of-interest *V*_0_ in study *S*_test_ using data from study *S*_train_ (also measuring *V*_0_), a prior random forest algorithm^39^ was modified to find variants in *S*_train_ whose *pre*-vac HAI predicts *V*_0_’s *post*-vac HAI. These random forests were then applied to predict *V*_0_‘s post-vac HAI in *S*_test_ (Supplementary Fig. S5a). More precisely, 50 decision trees were created, each randomly choosing five viruses with replacement (*V*_1_–*V*_5_, at least one must be *V*_0_, Methods). Each tree was internally trained on 30% of sera from study *S*_train_ and cross-validated on the remaining 70%. The 5 trees with the smallest cross-validation error comprised the final random forest model, with the geometric mean of their HAI outputs serving as *V*_0_‘s predicted post-vac titres in study *S*_test_ (Methods). This process was repeated for every combination of *S*_train_, *S*_test_, and *V*_0_.

In essence, this algorithm searched for the most predictive overlapping variants for *V*_0_. For example, the HAI of H3N2 A/Uruguay/716/2007 in the 2017 UGA cohort could be predicted by nine other vaccine studies that measured this variant, with each study estimating the post-vac HAI for all 271 individuals in the 2017 UGA study (Fig. 3b, more recent variants in Supplementary Fig. S5b). Taking the geometric mean of all nine values as the final prediction led to an RMSE = 2.3x (95% CI: 2.1–2.4x), comparable to the ≈2x error of the HAI assay. While pre-vac HAI for Uruguay 2007 was among the top two features for most trees, other historical or future variants had comparable importance (Supplementary Fig. S5c).

To predict a variant in any study, that variant must have been measured in at least one prior study. The considerable overlap in virus panels led to most viruses being predicted, even for recently emerged variants. For example, vaccine strains were predicted in 67% = 10/15 studies in Table 1, meaning that they were measured in a prior study before they became the vaccine strain.

### Using poor predictions to identify the factors that affect post-vaccination HAI

This compilation of random forests predicted the post-vac HAI of all overlapping variants between any pair of studies. Although we ultimately desired accurate predictions, poor estimates revealed which features fundamentally affect the antibody response in a complementary manner to Fig. 2. For each pair of vaccine studies, post-vac HAIs were predicted for all overlapping variants. The average prediction error for all subjects and all overlapping variants between two studies is shown by the transparency of the chord connecting these studies (poor accuracy shown with greater transparency; a few chords shown in Fig. 3c, all chords in Supplementary Fig. S6). While predictions were generally accurate, three datasets exhibited systematically poor predictions (with upper quartile RMSE > 4x, Supplementary Fig. S6).

The two earliest studies (1997–1998 Fonville) poorly predicted all subsequent vaccine studies (from 2009 or later). Unsurprisingly, this suggests that as antibody responses change over time, prior responses poorly predict outcomes too far into the future. This effect will be examined systematically in the following section.

The only other study with uniformly poor predictions was 2015 Hinojosa_*U*_, a study comprising children that self-reported as being uninfected and unvaccinated to influenza during the past five years. Notably, the 2014 and 2015 Hinojosa_*V*_ vaccine studies (children that were either infected or vaccinated in the past five years) had uniformly accurate predictions (interquartile RMSE between 2–3x, Supplementary Fig. S6), demonstrating that children can predict adult responses and vice versa. Instead, these results suggested that the unique exposure history of the children in 2015 Hinojosa_*U*_ led to their fundamentally different antibody responses. Since this was the only study with such exposure histories, it was dropped in the subsequent analysis. All remaining studies had upper quartile RMSE < 4x, so we next turn to the final step of determining how many prior seasons should be used to forecast future responses.

### This season’s vaccine responses are informed by responses from the past 10 seasons

The above framework gave pairwise predictions from study *S*_train_→*S*_test_. While predictions from a combination of studies (*S*_train,1_, *S*_train,2_, …)→*S*_test_ will likely be more robust, the number of possible training sets grows combinatorially; for example, the 2020 UGA study can be predicted using any combination of the 13 prior datasets (2^13^ ≈8000 choices).

For each study, the geometric mean of the post-vaccination response was computed from all vaccine studies conducted between 1 and *n* seasons beforehand (Fig. 3d). Prediction accuracy was maintained while using *n* ≤ 10 prior seasons, so the final algorithm combined the predictions of all vaccine studies from the past decade to forecast future antibody responses.

### Post-vaccination HAI is predicted with 2.4-fold error across 15 vaccine studies

The above framework uses a person’s pre-vac HAI to predict their response 1-month post-vac. As an example, forecasts of the vaccine strain (A/Hong Kong/4801/2014) in the 2017 UGA vaccine study demonstrate that while pre-vac HAI against the vaccine strain affects the response, the pre-vac HAI of variants can shift the vaccine strain response up to 10x (Supplementary Fig. S6d).

We next assessed these 2017 UGA predictions against the measured responses (results for all studies in Supplementary Fig. S7). Pre- and post-vac titres are shown for 5 (of 271) individuals, demonstrating the heterogeneity in both baseline HAI and the vaccine response (Fig. 3e). The RMSE across all 271 individuals is 2.3x (95% CI: 2.2–2.3x), comparable to the intrinsic noise of the HAI assay, and hence these predictions are as accurate as should be possible (Fig. 3e, IDs 004C, 175, 246, 081).

Notably, some individuals showed large consistent deviations (Fig. 3e, ID 226). To quantify this trend, predictions were extended across all studies to compute the deviation of the 5 worst-predicted variants for each serum. Overall, 9% of serum responses were substantially underpredicted (predictions ≥4x below measurements for 5 worst-predicted variants) and 3% were overpredicted (predictions ≥4x above measurements), while the remaining 88% of sera showed consistently small deviations across all variants (examples in Supplementary Fig. S7).

In addition to assessing each person’s response, every variant’s prediction was also evaluated. Returning to 2017 UGA, both the predicted and measured post-vac HAIs against the vaccine strain H3N2 A/Hong Kong/4801/2014 ranged from 40 to 640, resulting in a 2.4x prediction error (Fig. 3f, purple bars in Fig. 3e denote five such points). Post-vac HAIs were smaller for older variants, yet the distribution of titres showed a strong tendency to lie on the diagonal line that denotes accurate predictions (Fig. 3f, remaining variants in Supplementary Fig. S7).

**Fig. 4:**
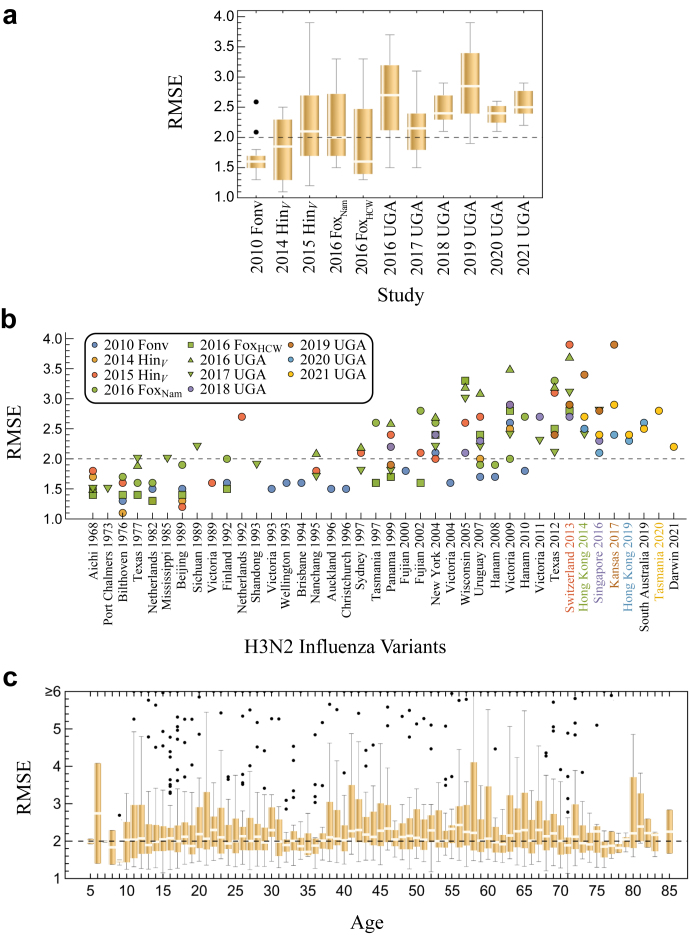
**Predicting vaccine responses across 11 datasets spanning 9 influenza seasons.** Distribution of root-mean-squared prediction error (RMSE) across each (a) study, (b) variant, or (c) age group. Coloured variants on the *x*-axis of Panel b denote vaccine strains for studies with the same colour (e.g., A/Hong Kong/4801/2014 was the vaccine strain for 2016–2017 studies). For each box plot, the horizontal line indicates the median, the box indicates the interquartile range, and the whiskers indicate 1.5 times the interquartile range.

Considering all variants in 2017 UGA, the RMSE for 15/18 = 83% were comparable to the ≈2x noise limit of the HAI assay (Fig. 3g). The final 3 variants had a larger RMSE ≈3x, still within the target range of ≤4x. Extending this analysis to all vaccine studies, 90% of variants were predicted with RMSE ≤ 3x while the remaining 10% had 3x < RMSE ≤ 4x. Notably, there was even minimal difference in RMSE across the 2017 and 2019–2021 seasons when the vaccine strain’s HA changed by ≥10aa (Fig. 4a and b). Prediction accuracy was stable across age groups, including the elderly responses (age ≥50) that have historically been harder to predict (Fig. 4c).^29^ Collectively, this represents 20,000 measurements with root-mean-square prediction error of 2.4x (95% CI: 2.34–2.40x).

The relatively small vaccine responses across all studies suggested that a null model (HAI_post_ = HAI_pre_) or a linear model (using each variant’s HAI_pre_ to predict its HAI_post_) may predict these data equally well. Across all 20,000 measurements, the linear and null models each had a larger prediction error (RMSE_linear_ = 3.0x, 95% CI_linear_: 2.98–3.12x; RMSE_null_ = 3.2x, 95% CI_null_: 3.12–3.21x) that was significantly larger than the random forest model (*p* < 0.002, one-sided permutation test). These differences were especially pronounced for more recent variants that tended to exhibit larger and more varied vaccine responses (grey points in Fig. 3g, Supplementary Fig. S8).

In addition, there were two major shortcomings for both the null and linear models. First, these models were more prone to large outliers. Whereas *all* variants were predicted with error ≤4x with the random forest approach, 16/133 = 12% of variants had error >4x (from 4.4–9.8x, *p* = 0.001 via a one-sided permutation test) with the null model, whereas 14/133 = 11% of variants had error >4x (from 4.2–52.9x, *p* = 0.006 via a one-sided permutation test) with the linear model (Supplementary Fig. S8). The null and linear models often exhibited these large errors for the vaccine strains, although both historical and future variants could elicit large errors. Notably, the linear model dramatically failed when the pre- to post-vac titres had a nearly vertical relation in a subset of studies, leading to prediction errors >100x (Supplementary Fig. S9). In comparison, the random forest approach led to slightly more accurate but far more robust predictions.

The second major failing of the linear and null models is that their coefficient of determination (*R*^2^) is consistently smaller than the random forest approach, especially for recent variants circulating within ∼10 years of the vaccine strain. For example, in the 2017 UGA study, 5/8 of the H3N2 variants circulating after 2005 achieved *R*^2^ < 0 with either the null or linear models, whereas the random forest had *R*^2^ between 0.2 and 0.6 (Supplementary Fig. S10).

As a further point of comparison, models trained within a single study (using 30% of subjects to predict the responses in the remaining 70%) always achieved median RMSE = 2.0–2.2x across any studies (Supplementary Fig. S11). While this represents a simpler prediction challenge than cross-study predictions forward in time, these results demonstrate that the ≈2x HAI noise limit is achievable and corroborates that the random forest predictions with this error are as accurate as possible.

### A prediction challenge across future seasons

The above analysis was carried out on all existing datasets, with predictions done forward in time (e.g., the 2020 vaccine study was predicted using the ∼40,000 measurements from 2019 and earlier, Table 1). To further demonstrate that prior vaccine studies can predict future responses, even in light of continual virus evolution, H3N2 responses 1-month post-vac were predicted in four new vaccine studies that we conducted (one in 2022, three in 2023, Fig. 5a). For this prediction challenge, the computational team (T.E.) was blinded by only receiving the pre-vac (day 0) HAIs to make the post-vac predictions.

**Fig. 5:**
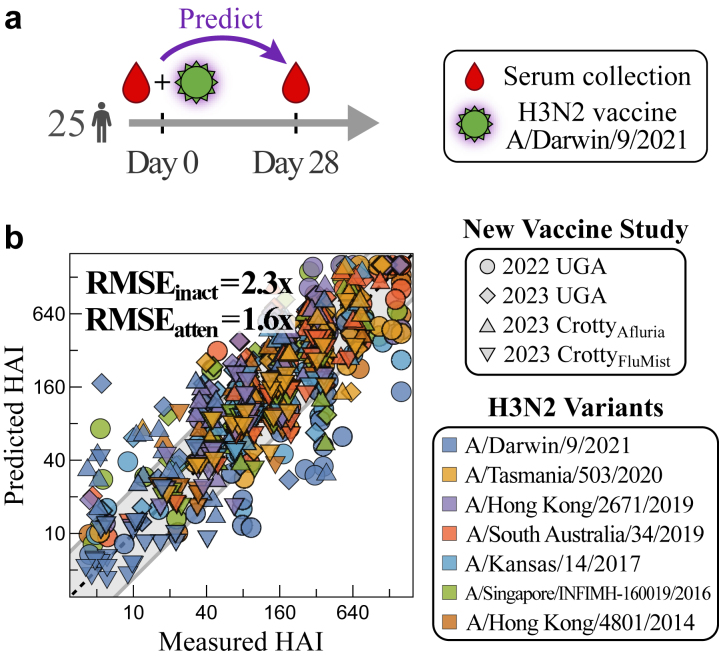
**Testing post-vaccination HAI predictions across new vaccine studies.** (a) Four new vaccine studies (2022 UGA, 2023 UGA, 2023 Crotty_Afluria_, 2023 Crotty_FluMist_) were carried out administering either the 2022 or 2023 quadrivalent influenza vaccine. Each study enrolled 25 participants, and post-vac sera were collected after 28 days and measured against a panel of H3N2 variants. (b) The predicted versus measured post-vac HAIs for all variants in all four studies (individual variant predictions in Supplementary Fig. S12). Error is shown for the three inactivated vaccine studies (RMSE_inact_ for 2022 UGA, 2023 UGA, and 2023 Crotty_Afluria_) and the live attenuated vaccine study (RMSE_atten_ for 2023 Crotty_FluMist_). The diagonal line *y* = *x* represents perfect predictions, while the grey bands denote 2.3-fold RMSE for the inactivated predictions.

Two of these vaccine studies (2022 UGA and 2023 UGA) followed the same format as the prior UGA cohorts,^43^ administering the inactivated Fluzone vaccine to participants in Athens GA (demographics in Supplementary Fig. S1). The other two studies administered either Afluria or FluMist in San Diego CA (2023 Crotty_Afluria_ and Crotty_FluMist_). All four studies enrolled 25 participants and administered the seasonal vaccine containing the 2022 and 2023 H3N2 vaccine strain A/Darwin/9/2021. Each study measured HAI against this vaccine strain as well as six historical variants (A/Hong Kong/4801/2014, A/Singapore/INFIMH-160019/2016, A/Kansas/14/2017, A/South Australia/34/2019, A/Hong Kong/2671/2019, and A/Tasmania/503/2020) that were chosen to overlap with the 2020 and 2021 UGA studies.

Unlike inactivated vaccines, the live attenuated vaccine FluMist has no known correlates of protection. The one other example in Table 1 using FluMist found no increase in post-vac serum HAI.^41^ More generally, some studies have reported that live attenuated vaccines elicit little-to-no HAI responses,^57^, ^58^, ^59^, ^60^ others have found HAI increases in children <18 years old,^61^, ^62^, ^63^, ^64^ and a randomized double-blind clinical study found a very small HAI rise of 1.05-fold against H3N2 in adults.^65^ Since the Crotty_FluMist_ cohort comprised individuals >18 years old, predictions were made using the null model that assumed each variant’s post-vac HAI equals its pre-vac titre. In contrast, the inactivated vaccines Fluzone and Afluria were predicted using the random forest algorithm described above.

Collectively, random forests predicted the three inactivated vaccine studies [2022 UGA, 2023 UGA, 2023 Crotty_Afluria_] with RMSE = 2.35x (95% CI: 2.22–2.48x) (Fig. 5b), outperforming the null model with near-significance (RMSE = 2.58x, 95% CI: 2.38–2.80x; *p* = 0.06, one-sided permutation test) and the linear model with significance (RMSE = 2.62x, 95% CI: 2.46–2.79x; *p* = 0.01, one-sided permutation test). The largest prediction error occurred for the vaccine strain Darwin 2021 (RMSE = 2.7–4.4x), while the remaining variants had ≲2x error (Supplementary Fig. S12).

Live attenuated predictions for 2023 Crotty_FluMist_ were better for the null model (RMSE = 1.6x, 95% CI: 1.48–1.68x) than the linear model (RMSE = 2.3x, 95% CI: 2.17–2.49x, Supplementary Fig. S12). The null model was consistently within the noise threshold of the HAI assay (<2x), confirming that HAI titres do not noticeably increase in adults receiving this live attenuated vaccine.

As before, each vaccine study was predicted forward in time (e.g., models of the 2023 UGA and 2023 Crotty_Afluria_ vaccines were trained on vaccine studies from 2022 and earlier). However, since the 2023 UGA study was carried out in September while the 2023 Crotty_Afluria_ study was done in December, the former study could augment the latter's predictions. Rather than training on all vaccine studies from the last 10 years, models were trained using every subset of these studies, and the subset yielding the best predictions on 2023 UGA was used to predict the 2023 Crotty_Afluria_ responses. These specialised predictions led to a small but not significant improvement in the Darwin 2021 vaccine strain predictions (RMSE = 3.5 → 3.0x, *p* = 0.2 from one-sided permutation test) and minimally improved the other variants (Supplementary Fig. S12e).

### Using the model to recoup known phenomena and discover predictive features of vaccine responses

The model provides an opportunity to search for general relationships between an individual’s pre- and post-vaccination HAI. Few such rules are known, with one major exception being the antibody ceiling effect where the subjects with a large pre-vac titre hit a “ceiling” and exhibit smaller fold-change post-vac. Other results have been noted in individual studies; for example, the 2016 Fox_Nam_ study found that individuals recently infected by H3N2 have higher fold-change than those with no recent exposure.^42^

Our model replicated these vaccine responses to an extent. The recently infected subjects in 2016 Fox_Nam_ were predicted to have 1.3x larger fold-change (comparable to 1.5x found experimentally) than subjects with no prior infection (Supplementary Fig. S13a). Little-to-no antibody ceiling was both measured and predicted for older variants circulating before 2000. For more recent variants, the model predicted a 2x reduction in a variant’s fold-change for each 34x increase in its pre-vac HAI (while experiments found that only a 10x increase in pre-vac HAI is needed, Supplementary Fig. S13b). This effect is small, as increasing pre-vac HAI from 10 → 320 (the range of most pre-vac titres for these viruses) is predicted to blunt fold-change by 2x (while experiments report a 2.8x blunting). Thus, the model recapitulates these phenomena, although with smaller effect sizes.

**Fig. 6:**
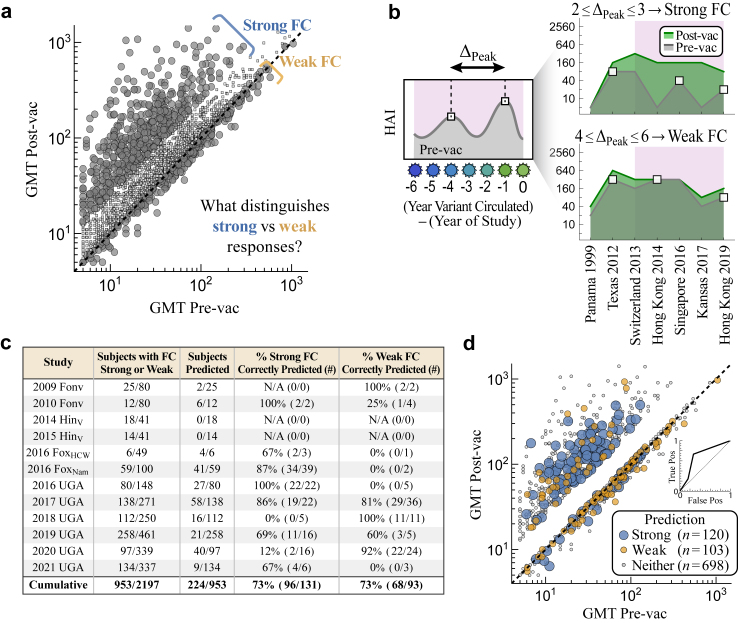
**Time between HAI peaks of recent influenza variants distinguishes strong from weak fold-change responses.** (a) Vaccine responses were defined as strong fold-change (FC ≡ post-vac GMT/pre-vac GMT ≥ 2.5) or weak fold-change (FC ≤ 1.1) across all variants, in contrast to Fig. 1 analysing absolute post-vac HAI. Intermediate FCs were not considered. (b) Δ_Peak_ was defined as the years between the two most recent HAI peaks (white squares) for variants circulating 0–6 years prior to the study (pink region, Methods). Plateaus containing equally strong variants have their peak at the earlier variant. Subjects were classified as strong if 2 ≤ Δ_Peak_ ≤ 3 and weak if 4 ≤ Δ_Peak_ ≤ 6; subjects with longer Δ_Peak_ or ≤2 peaks within this time period were not classified. Data shown for IDs 208 [top right] and 50C [bottom right] from the 2019 UGA dataset. (c) Classification accuracy across all studies. (d) Classifications mapped back onto the pre- and post-vac GMTs for the strong and weak fold-change responses across all studies. Inset: Receiver operating characteristic analysis. Fraction of false positives (*x*-axis, 1-fraction weak correctly identified) and true positives (*y*-axis, fraction strong responders correctly identified) for different Δ_Peak_ cutoffs between weak/strong phenotypes.

Given this caveat, we implemented a robust search for pre-vaccination features that distinguish strong fold-change responses (defined as GMT_post_ ≥ 2.5·GMT_pre_ against the full variant panel) from weak responses (GMT_post_ ≤ 1.1·GMT_pre_, Fig. 6a), while accounting for potential prediction error and experimental error. Note that unlike the prior sections analysing absolute HAI, the following analysis uses fold-change (FC) pre-to-post vaccination, often deemed to be an equally important metric of the response.

To account for potential model and experimental error, we assessed which subjects not only exhibited either a strong/weak FC response, but where small perturbations (increasing or decreasing any variant’s pre-vac HAI by 2x) were predicted to elicit this same strong/weak FC ≥ 75% of the time (Supplementary Fig. S14 and S15). The resulting “robustly strong” and “robustly weak” pre-vac states were further validated using a support vector machine and nearest neighbour classification trained on the strong/weak FC responses in each dataset and then used to classify all perturbed states (Methods).

The robustly strong HAI responses were noticeably more jagged than the robustly weak responses, especially against recent variants (Supplementary Fig. S14). The time Δ_Peak_ between the two most recent pre-vac HAI peaks correlated with post-vac fold-change (with peaks defined using the dominant variants circulating within 6 years of each study, plateaus marked at the older variant, Methods). 73% of strong responders had a gap of Δ_Peak_ = 2–3 years while 73% of weak responses had a larger gap of 4–6 years (Fig. 6b–d), with a significant decrease in Δ_Peak_ between both classes (*p* < 0.001, one-sided permutation test). To assess the sensitivity of the threshold separating weak versus strong responses, the fraction of strong and weak responders correctly predicted was assessed for different thresholds (Fig. 6d inset; strong if Δ_Peak_ = 2 and weak if Δ_Peak_ = 3–6, strong if Δ_Peak_ = 2–3 and weak if Δ_Peak_ = 4–6, …). The area under this curve was 0.7, with the thresholds used above yielding the best classification accuracy.

As a point of comparison, using age ≤ 65 or pre-vac GMT ≤ 80 as an indication of weak-fold change tends to accurately predict strong responses (83–92% accuracy) at the cost of characterising weak responses (17–37% accuracy, Supplementary Fig. S16). In summary, the periodicity of pre-vac HAI against recent variants predicts the fold-change of the post-vaccination response, and such features should be considered in conjunction with features such as age or pre-vaccination titres.

## Discussion

This work developed a computational framework that uses an individual’s pre-vaccination HAI to forecast their response 1-month post-vaccination. The model is exclusively trained on prior seasons, with each prediction forecasting the response in a never-before-seen season. Any such model must be able to handle the heterogeneous immune responses across the population, updates to the vaccine composition, and the myriad differences in study designs.

Our approach used: (1) pre-vac HAI against the vaccine strain and (2) pre-vac HAI against historical variants, both of which improved prediction by ∼30% relative to predictions based solely on demographic features such as age. While many studies measure the vaccine strain, data from variants is less common, yet recent work showed that variants can reveal long-term trends in the antibody response^66^ or estimate protection against future variants.^67^

In the context of vaccine responses, measuring variants offers three main advantages. First, prior work showed that titres from variants can infer exposure history,^30^^,^^68^ a feature that is known to affect the antibody response yet is difficult to accurately report. Conceptually, variants break up potential degeneracies in the data: for example, two subjects may have the same pre-vac HAI = 20, but in one case it was caused by a minor recent infection while in the other case it was caused by a larger infection several years back. Measuring the response against historical variants would distinguish these two scenarios, and future work should extend this approach to determine if variant data are useful in other viral contexts.

Second, variants provide common features that can be used for prediction. Even the oldest 1997 Fonville study had ≥4 overlapping variants with every other vaccine study we considered from 1998 to 2018, letting us test how far forward in time the patterns in early studies continue to hold.

Third, previous work showed that measuring a serum’s pre- or post-vac HAI against as few as 4 variants can predict other variants’ HAI at this same time point (e.g., one pre-vac response can predict another, or that post-vac can predict post-vac).^39^ This demonstrated that at each slice of time, there are conserved patterns of cross-reactivity between variants that hold across datasets, and in this work we pushed further by using variants to predict the pre-to-post vaccine response.

In comparison to variant pre-vac HAI, a person’s age, sex, vaccine dose, or geographic location led to smaller improvements in prediction accuracy. These same trends held in our four new vaccine studies (99 subject total, all from the US), further corroborating the 15 prior vaccine studies that had a greater range of demographics and geographic locations. Note that other factors that could further affect HAI such as genetics,^27^^,^^52^ nutrition,^69^^,^^70^ CMV status,^71^^,^^72^ or other comorbidities^73^^,^^74^ were not measured in these studies and hence not considered.

Across the measured features, the lack of predictive power is especially surprising for age, which is known to affect the influenza antibody response.^33^^,^^49^^,^^51^^,^^75^ One explanation is that these age effects may be small (as shown for the infection history and antibody ceiling effects), or they may be dataset-specific and change across seasons. Yet while prior studies found that influenza responses are harder to predict in the elderly,^29^ we found uniformly good predictions across 5–85 year olds.

Indeed, children's responses (2014–2015 Hinojosa_V_) accurately predicted adult responses and vice versa (upper quartile RMSE < 4x). Aside from the two oldest studies, the only poorly predicted dataset was 2015 Hinojosa_*U*_, a cohort containing subjects that self-reported as being uninfected and unvaccinated in the past five years.^41^ Notably, even the 2016 Fox_Nam_ cohort comprising adults receiving the first influenza vaccine of their life was well-predicted. Taken together, these results suggest that a lack of recent influenza infections (and possibly young age) can result in fundamentally different antibody responses, and future work should examine whether cohorts with no recent influenza exposure can better predict one another.

If these results translate into animal studies, they suggest that experiments on naive mice or ferrets may have limited transferability to humans not only because they are a different species, but also because of their non-existent immune history. This hypothesis can be tested by comparing the vaccine response in humans to that in mice/ferrets with different exposure histories.^76^, ^77^, ^78^, ^79^

Since most influenza deaths occur in the elderly (age 65+), antibody responses in this age group are particularly important. In the UGA studies, all elderly individuals ≥65 years old in the UGA studies were offered high-dose Fluzone, and 76% (=259/340) received it (see the GitHub dataset for each person’s vaccine dose). However, we found no stark difference between the HAIs of elderly individuals (age 65+) receiving high-dose vaccines and an adjacent age group (55–64) receiving standard dose Fluzone in any season. Notably, multiple prior studies found that high-dose vaccines elicited higher HAI or led to fewer hospitalizations,^80^, ^81^, ^82^ although some studies found little effect.^83^ In the UGA studies, the high-dose vaccines may have partially blunted differences between these elderly age groups.

Few methods exist to predict the post-vac response from pre-vac data. Antigenic cartography uses naive ferret data to predict the relationship between the HAI of each strain,^4^^,^^67^ but it cannot predict the magnitude of the response to identify weak versus strong responders. The null and linear models introduced here can predict responses from multiple variants, yet both were more prone to large prediction errors and explain little-to-no variance in post-vac HAI for variants circulating within ∼10 years of the vaccine strain. Vaccine inertia demonstrates that the prior season’s response can qualitatively inform whether a person’s response in the current season will be under or over some HAI threshold, and future work will examine whether such patterns hold across multiple seasons.

Rather than predicting absolute titres, other methods have predicted adjacent tasks including: 1) is the vaccine strain’s HAI above or below a threshold [e.g., post-vac HAI ≥40 and/or fold-change ≥4x],^24^^,^^26^ 2) what is the average or maximum titre across the H1N1, H3N2, and influenza B components of the vaccine,^27^ or 3) how accurately can normalized titres be predicted.^29^^,^^36^ The challenge in this work is a superset of those approaches from which thresholds or normalized responses can be inferred. Future work will explore whether H1N1 and influenza B vaccine responses can be similarly predicted, and whether responses from pre-2009 pandemic H1N1 variants predict post-pandemic responses.

Previous efforts found that 13 different vaccines (including influenza) can induce universal signatures without needing to specify the vaccine antigen.^29^ We similarly did not directly encode information about the vaccine strain, but rather indirectly learned such information in a data-driven manner by training on all studies from the past 10 seasons. This crude encoding cannot distinguish between vaccine formulations or even vaccine strain composition, and hence the overall 2.4x prediction error across all studies suggests that formulation and vaccine strain have a minor effect on the resulting vaccine response. This holds across all seasons, including cases where the H3N2 vaccine strain stayed the same (2016→2017, 2022→2023) or changed across clades (2018→2019 [Kansas 2017]→2020). Had a different H3N2 variant been chosen for the 2019 vaccine, we hypothesize that vaccine studies would have looked similar (with the new vaccine strain replacing Kansas 2017).

While most of the studies we analysed administered Fluzone, other inactivated vaccines (Afluria, Fluarix, Vaxigrip) were predicted with uniformly low error, suggesting that each formulation acts similarly. The only notable exception was the live attenuated vaccine FluMist that elicited no antibody response in 84% of individuals (pre-vac HAI ≈ post-vac HAI for all variants), and future work will determine where other vaccine types (e.g., recombinant, adjuvanted) fall along this spectrum. Note that while inactivated vaccines induced a measurable antibody response on average, ∼35% of individuals still exhibited little-to-no response (≤2x fold-change against any variant).

Across the influenza seasons with predictions (2009–2010, 2014–2023), our approach characterises ∼88% of sera at 1-month post-vac within the roughly 2x error of the HAI assay. Yet the most extreme responses—those with the very largest and very smallest titres—were poorly predicted, likely because the vaccines elicited a small response in most individuals which skewed models to predict small responses. The 3% of overpredicted sera may represent individuals infected in the month prior to vaccination, edge cases poorly handled by the model, or experimental error. Supporting the prior infection hypothesis, these individuals had higher pre-vac HAI against their vaccine strain (GMT = 96 in overpredicted sera, GMT = 35 across remaining sera). The 9% of underpredicted responses, comparable to the ∼10% annual influenza incidence,^84^ may similarly represent breakthrough infections that magnified the HAI response or a shortcoming of the model. In support of the breakthrough infection hypothesis, these individuals exhibited a larger backboost in fold-change across prior variants (geometric mean fold-change = 6.5x across all variants in underpredicted sera, compared to 1.7x across remaining sera) expected from infections.

Yet a model need not perfectly predict all data to be useful. Individuals robustly categorized as strong responders, even when their pre-vac titres were perturbed by 2x, exhibited more jagged HAI landscapes, and a classification scheme based on this feature had 73% accuracy. In comparison, a recent study distinguished the strongest and weakest influenza vaccine responses with 63% accuracy,^29^ and to our knowledge, any other method with greater accuracy took on a different prediction task (e.g., predicting post-vac HAI ≥ 40^28^ or vaccine breadth^23^).

This jaggedness or short-term periodicity in HAI titres serves as a counterpoint to the long-term periodicity found in a recent cross-sectional study,^66^ and it is unclear what mechanisms underlie either behaviour. We speculate that the oscillatory HAIs from robustly strong responders imply that their antibodies target variant-specific epitopes, and hence they are prone to respond strongly to drifted vaccine strains. Conversely, robustly weak responders may target more conserved epitopes that better inhibit future strains, which partially blocks their response to the next few vaccines they encounter. If this is true, then future vaccines may need to be specifically designed for robustly weak responders to overcome their preexisting immunity.

One limitation of this study is that we only predicted the peak response 1-month post-vaccination without examining long-term antibody waning. However, recent work suggests that these two regimes are tightly connected, where individuals with fold-change ≤8x at 1-month returning to baseline within 180 days while those with fold-change ≥16x maintain an elevated response out to 1-year post-vaccination.^85^ Hence, the predictions for a single time point should help determine the long-term antibody trajectory.

Another limitation is that roughly half the studies examined used the Fluzone vaccine, and future work should examine other vaccine formulations in additional seasons against more diverse populations to test its generality. To maximize clinical applications, such models should only use pre-vac titres and responses from prior seasons to predict subsequent responses, shifting from a descriptive to a predictive mindset.

The improved predictions from measuring multiple variants underscores the potential to integrate other aspects of the immune response (antibody effector functions,^86^ cellular or innate immunity,^87^^,^^88^ functional responses^89^) or virus factors (sequences/glycosylation of HA,^90^ incorporating other viral proteins^12^) for further accuracy. The egg-grown vaccines considered here may have adaptations that lead to different cross-reactivity relations than those found in circulating viruses. While HAI quantifies the action of the entire antibody repertoire, a refined understanding of how plasmablasts and memory B cells target specific epitopes is needed to quantify susceptibility to escape mutations.

Each vaccine study requires substantial time and effort, but the sera collected have utility beyond quantifying the vaccine response in a single season. For example, existing sera can be tested against next year’s vaccine strain, or against a newly emerged variant, to infer their inhibition during an upcoming season. Alternatively, vaccine responses from early in the season or from southern hemisphere studies (whose influenza season is offset by 6 months) could augment predictions and inform strain selection in the northern hemisphere.

The central goal of influenza vaccines is to elicit a robust antibody response against circulating strains and potential escape mutants. This work suggests that measuring responses to historic variants helps predict such responses, but future efforts should focus on measuring and predicting the antibody response—including neutralization responses from cell-grown vaccines—against this swath of recent variants.

Predicting influenza vaccine responses at the start of each season sets the stage to design better vaccine strategies. If we could estimate the durability of each person’s response, individuals whose HAI titres rapidly decay could get booster doses. If a person’s response to multiple vaccine options can be predicted at the start of a season, we could recommend the best vaccine for their specific immune state. Next generation vaccines under development will enable more complex vaccine strategies, and while a universal one-size-fits-all vaccination scheme may ultimately result, it is also worth considering vaccine recommendation schemes that better utilise the tools at hand.

## Contributors

Conceptualization: TE. Data acquisition: HS, MAC, JDA, HBH, TE. Data verification: HS, TMR, TE. Formal analysis: TE. Writing—original draft: TE. Writing—review & editing: HS, MAC, JDA, SC, TMR, TE.

## Data sharing statement

The data and code used in this work are contained in GitHub (https://github.com/TalEinav/PredictFluVaccine). Both the raw data and random forest predictions are provided in a CSV file. We also provide a supplementary Mathematica notebook that implements our prediction framework and reproduces all figures from this manuscript.

## Declaration of interests

SC has consulted for GSK, JP Morgan, Citi, Morgan Stanley, Avalia NZ, Nutcracker Therapeutics, University of California, California State Universities, United Airlines, Adagio, Sanofi, and Roche on infectious diseases. All other authors declare no competing interests.

## References

[1] Sun H., Yang J., Zhang T. (2013). Using sequence data to infer the antigenicity of influenza virus. mBio.

[2] Petrova V.N., Russell C.A. (2018). The evolution of seasonal influenza viruses. Nat Rev Microbiol.

[3] Morris D.H., Gostic K.M., Pompei S. (2018). Predictive modeling of influenza shows the promise of applied evolutionary biology. Trends Microbiol.

[4] Fonville J.M., Wilks S.H., James S.L. (2014). Antibody landscapes after influenza virus infection or vaccination. Science.

[5] Cobey S., Hensley S.E. (2017). Immune history and influenza virus susceptibility. Curr Opin Virol.

[6] McLean H.Q., Thompson M.G., Sundaram M.E. (2014). Impact of repeated vaccination on vaccine effectiveness against influenza A(H3N2) and B during 8 seasons. Clin Infect Dis.

[7] Lewnard J.A., Cobey S. (2018). Immune history and influenza vaccine effectiveness. Vaccines.

[8] Jang H., Ross T.M. (2019). Preexisting influenza specific immunity and vaccine effectiveness. Expert Rev Vaccines.

[9] Auladell M., Phuong H.V.M., Mai L.T.Q. (2022). Influenza virus infection history shapes antibody responses to influenza vaccination. Nat Med.

[10] Zhao X., Fang V.J., Ohmit S.E., Monto A.S., Cook A.R., Cowling B.J. (2016). Quantifying protection against influenza virus infection measured by hemagglutination-inhibition assays in vaccine trials. Epidemiology.

[11] Cowling B.J., Lim W.W., Perera R. (2019). Influenza hemagglutination-inhibition antibody titer as a mediator of vaccine-induced protection for influenza B. Clin Infect Dis.

[12] Krammer F. (2019). The human antibody response to influenza A virus infection and vaccination. Nat Rev Immunol.

[13] Coudeville L., Bailleux F., Riche B., Megas F., Andre P., Ecochard R. (2010). Relationship between haemagglutination-inhibiting antibody titres and clinical protection against influenza: development and application of a bayesian random-effects model. BMC Med Res Methodol.

[14] Allison M.A., Daley M.F., Crane L.A. (2006). Influenza vaccine effectiveness in healthy 6- to 21-month-old children during the 2003-2004 season. J Pediatr.

[15] Rudenko L., Naykhin A., Donina S. (2015). Assessment of immune responses to H5N1 inactivated influenza vaccine among individuals previously primed with H5N2 live attenuated influenza vaccine. Hum Vaccin Immunother.

[16] Lu X., Liu F., Tzeng W.-P., York I.A., Tumpey T.M., Levine M.Z. (2024). Antibody-mediated suppression regulates the humoral immune response to influenza vaccination in humans. J Infect Dis.

[17] Goodwin E., Gibbs J.S., Yewdell J.W., Eisenlohr L.C., Hensley S.E. (2024). Influenza virus antibodies inhibit antigen-specific *de novo* B cell responses in mice. J Virol.

[18] Lessler J., Riley S., Read J.M. (2012). Evidence for antigenic seniority in influenza A (H3N2) antibody responses in southern China. PLoS Pathog.

[19] Yewdell J.W., Santos J.J.S. (2021). Original antigenic sin: how original? How sinful?. Cold Spring Harb Perspect Med.

[20] Oidtman R.J., Arevalo P., Bi Q. (2021). Influenza immune escape under heterogeneous host immune histories. Trends Microbiol.

[21] Brouwer A.F., Balmaseda A., Gresh L. (2022). Birth cohort relative to an influenza A virus's antigenic cluster introduction drives patterns of children's antibody titers. PLoS Pathog.

[22] Goronzy J.J., Fulbright J.W., Crowson C.S., Poland G.A., O'Fallon W.M., Weyand C.M. (2001). Value of immunological markers in predicting responsiveness to influenza vaccination in elderly individuals. J Virol.

[23] Furman D., Jojic V., Kidd B. (2013). Apoptosis and other immune biomarkers predict influenza vaccine responsiveness. Mol Syst Biol.

[24] Forst C.V., Chung M., Hockman M. (2022). Vaccination history, body mass index, age, and baseline gene expression predict influenza vaccination outcomes. Viruses.

[25] Parvandeh S., Poland G.A., Kennedy R.B., McKinney B.A. (2019). Multi-level model to predict antibody response to influenza vaccine using gene expression interaction network feature selection. Microorganisms.

[26] Wu S., Ross T.M., Carlock M.A., Ghedin E., Choi H., Vogel C. (2022). Evaluation of determinants of the serological response to the quadrivalent split-inactivated influenza vaccine. Mol Syst Biol.

[27] Nakaya H.I., Hagan T., Duraisingham S.S. (2015). Systems analysis of immunity to influenza vaccination across multiple years and in diverse populations reveals shared molecular signatures. Immunity.

[28] Jurchott K., Schulz A.R., Bozzetti C. (2016). Highly predictive model for a protective immune response to the A(H1N1)pdm2009 influenza strain after seasonal vaccination. PLoS One.

[29] Fourati S., Tomalin L.E., Mulè M.P. (2022). Pan-vaccine analysis reveals innate immune endotypes predictive of antibody responses to vaccination. Nat Immunol.

[30] Kucharski A.J., Lessler J., Cummings D.A.T., Riley S. (2018). Timescales of influenza A/H3N2 antibody dynamics. PLoS Biol.

[31] Kosikova M., Li L., Radvak P., Ye Z., Wan X.F., Xie H. (2018). Imprinting of repeated influenza A/H3 exposures on antibody quantity and antibody quality: implications for seasonal vaccine strain selection and vaccine performance. Clin Infect Dis.

[32] Gostic K.M., Ambrose M., Worobey M., Lloyd-Smith J.O. (2016). Potent protection against H5N1 and H7N9 influenza via childhood hemagglutinin imprinting. Science.

[33] Vinh D.N., Nhat N.T.D., De Bruin E. (2021). Age-seroprevalence curves for the multi-strain structure of influenza A virus. Nat Commun.

[34] Avey S., Mohanty S., Chawla D.G. (2020). Seasonal variability and shared molecular signatures of inactivated influenza vaccination in young and older adults. J Immunol.

[35] Diray-Arce J., Miller H.E.R., Henrich E. (2022). The Immune Signatures data resource, a compendium of systems vaccinology datasets. Scientific Data.

[36] Tsang J.S., Schwartzberg P.L., Kotliarov Y. (2014). Global analyses of human immune variation reveal baseline predictors of postvaccination responses. Cell.

[37] Cortese M., Sherman A.C., Rouphael N.G., Pulendran B. (2021). Systems biological analysis of immune response to influenza vaccination. Cold Spring Harb Perspect Med.

[38] Einav T., Cleary B. (2022). Extrapolating missing antibody-virus measurements across serological studies. Cell Syst.

[39] Einav T., Ma R. (2023). Using interpretable machine learning to extend heterogeneous antibody-virus datasets. Cell Rep Methods.

[40] Hodgson D., Sánchez-Ovando S., Carolan L. (2025). Quantifying the impact of pre-vaccination titre and vaccination history on influenza vaccine immunogenicity. Vaccine.

[41] Hinojosa M., Shepard S.S., Chung J.R. (2021). Impact of immune priming, vaccination, and infection on influenza A(H3N2) antibody landscapes in children. J Infect Dis.

[42] Fox A., Carolan L., Leung V. (2022). Opposing effects of prior infection versus prior vaccination on vaccine immunogenicity against influenza A(H3N2) viruses. Viruses.

[43] Carlock M.A., Allen J.D., Hanley H.B., Ross T.M. (2024). Longitudinal assessment of human antibody binding to hemagglutinin elicited by split-inactivated influenza vaccination over six consecutive seasons. PLoS One.

[44] Harvey W.T., Davies V., Daniels R.S. (2023). A Bayesian approach to incorporate structural data into the mapping of genotype to antigenic phenotype of influenza A(H3N2) viruses. PLoS Comp Biol.

[45] Hobson D., Curry R.L., Beare A.S., Ward-Gardner A. (1972). The role of serum haemagglutination-inhibiting antibody in protection against challenge infection with influenza A2 and B viruses. Epidemiol Infect.

[46] Paccalin M., Plouzeau C., Bouche G. (2006). Lack of correlation between nutritional status and seroprotection against influenza in a long term care facility. Scand J Infect Dis.

[47] Black S., Nicolay U., Vesikari T. (2011). Hemagglutination inhibition antibody titers as a correlate of protection for inactivated influenza vaccines in children. Pediatr Infect Dis J.

[48] Hay J.A., Zhu H., Jiang C.Q. (2024). Reconstructed influenza A/H3N2 infection histories reveal variation in incidence and antibody dynamics over the life course. PLoS Biol.

[49] Henry C., Zheng N.Y., Huang M. (2019). Influenza virus vaccination elicits poorly adapted B cell responses in elderly individuals. Cell Host Microbe.

[50] Gouma S., Kim K., Weirick M.E. (2020). Middle-aged individuals may be in a perpetual state of H3N2 influenza virus susceptibility. Nat Commun.

[51] Welsh F.C., Eguia R.T., Lee J.M. (2024). Age-dependent heterogeneity in the antigenic effects of mutations to influenza hemagglutinin. Cell Host Microbe.

[52] Avey S., Cheung F., Fermin D. (2017). Multicohort analysis reveals baseline transcriptional predictors of influenza vaccination responses. Sci Immunol.

[53] Andrews S.F., Huang Y., Kaur K. (2015). Immune history profoundly affects broadly protective B cell responses to influenza. Sci Transl Med.

[54] Hopping A.M., McElhaney J., Fonville J.M., Powers D.C., Beyer W.E.P., Smith D.J. (2016). The confounded effects of age and exposure history in response to influenza vaccination. Vaccine.

[55] Loes A.N., Tarabi R.A.L., Huddleston J. (2024). High-throughput sequencing-based neutralization assay reveals how repeated vaccinations impact titers to recent human H1N1 influenza strains. J Virol.

[56] Koel B.F., Burke D.F., Bestebroer T.M. (2013). Substitutions near the receptor binding site determine major antigenic change during influenza virus evolution. Science.

[57] Barría M.I., Garrido J.L., Stein C. (2013). Localized mucosal response to intranasal live attenuated influenza vaccine in adults. J Infect Dis.

[58] Williams K.V., Zhai B., Alcorn J.F. (2022). A randomized controlled trial of antibody response to 2019–20 cell-based inactivated and egg-based live attenuated influenza vaccines in children and young adults. Vaccine.

[59] Yegorov S., Celeste D.B., Gomes K.B. (2022). Inactivated and live-attenuated seasonal influenza vaccines boost broadly neutralizing antibodies in children. Cell Rep Med.

[60] Meade P., Strohmeier S., Bermúdez-González M.C. (2023). Antigenic landscape analysis of individuals vaccinated with a universal influenza virus vaccine candidate reveals induction of cross-subtype immunity. J Virol.

[61] Hoft D.F., Babusis E., Worku S. (2011). Live and inactivated influenza vaccines induce similar humoral responses, but only live vaccines induce diverse T-cell responses in young children. J Infect Dis.

[62] Block S.L., Falloon J., Hirschfield J.A. (2012). Immunogenicity and safety of a quadrivalent live attenuated influenza vaccine in children. Pediatr Infect Dis J.

[63] Manenti A., Tete S.M., Mohn K.G.I. (2017). Comparative analysis of influenza A(H3N2) virus hemagglutinin specific IgG subclass and IgA responses in children and adults after influenza vaccination. Vaccine.

[64] Ertesvag N.U., Cox R.J., Lartey S.L., Mohn K.G., Brokstad K.A., Trieu M.C. (2022). Seasonal influenza vaccination expands hemagglutinin-specific antibody breadth to older and future A/H3N2 viruses. NPJ Vaccin.

[65] Block S.L., Yi T., Sheldon E., Dubovsky F., Falloon J. (2011). A randomized, double-blind noninferiority study of quadrivalent live attenuated influenza vaccine in adults. Vaccine.

[66] Yang B., Garcia-Carreras B., Lessler J. (2022). Long term intrinsic cycling in human life course antibody responses to influenza A(H3N2): an observational and modeling study. eLife.

[67] Meijers M., Ruchnewitz D., Eberhardt J., Karmakar M., Łuksza M., Lässig M. (2025).

[68] Kucharski A.J., Lessler J., Read J.M. (2015). Estimating the life course of influenza A(H3N2) antibody responses from cross-sectional data. PLoS Biol.

[69] Hara M., Tanaka K., Hirota Y. (2005). Immune response to influenza vaccine in healthy adults and the elderly: association with nutritional status. Vaccine.

[70] Eskenazi B., Rauch S., Elsiwi B. (2025). Undernutrition and antibody response to measles, tetanus and Haemophilus Influenzae type b (Hib) vaccination in pre-school south African children: the VHEMBE birth cohort study. Vaccine.

[71] Davis M.M., Brodin P. (2018). Rebooting human immunology. Annu Rev Immunol.

[72] Van Den Berg S.P.H., Wong A., Hendriks M., Jacobi R.H.J., Van Baarle D., Van Beek J. (2018). Negative effect of age, but not of latent cytomegalovirus infection on the antibody response to a novel influenza vaccine strain in healthy adults. Front Immunol.

[73] Wiggins K.B., Smith M.A., Schultz-Cherry S. (2021). The nature of immune responses to influenza vaccination in high-risk populations. Viruses.

[74] Kimball J., Zhu Y., Wyatt D., Trabue C.H., Talbot H.K. (2021). Influenza vaccine failure associated with age and immunosuppression. J Infect Dis.

[75] Kim K., Marcos Gouma S., Madison Scott, Cobey S. (2024). Measures of population immunity can predict the dominant clade of influenza A (H3N2) in the 2017–2018 season and reveal age-associated differences in susceptibility and antibody-binding specificity. Influenza Other Respir Viruses.

[76] Nachbagauer R., Choi A., Hirsh A. (2017). Defining the antibody cross-reactome directed against the influenza virus surface glycoproteins. Nat Immunol.

[77] Allen J.D., Ross T.M. (2021). Evaluation of next-generation H3 influenza vaccines in ferrets pre-immune to historical H3N2 viruses. Front Immunol.

[78] Uno N., Ross T.M. (2024). Multivalent next generation influenza virus vaccines protect against seasonal and pre-pandemic viruses. Sci Rep.

[79] Ge Y., Lu Y., Allen J.D. (2024). Pre-existing immunity to influenza aids ferrets in developing stronger and broader H3 vaccine-induced antibody responses. Vaccine.

[80] DiazGranados C.A., Dunning A.J., Kimmel M. (2014). Efficacy of high-dose versus standard-dose influenza vaccine in older adults. NEJM.

[81] Sanchez L., Nakama T., Nagai H. (2023). Superior immunogenicity of high-dose quadrivalent inactivated influenza vaccine versus Standard-Dose vaccine in Japanese Adults ≥60 years of age: results from a phase III, randomized clinical trial. Vaccine.

[82] Johansen N.D., Modin D., Skaarup K.G. (2024). Effectiveness of high-dose versus standard-dose quadrivalent influenza vaccine against recurrent hospitalizations and mortality in relation to influenza circulation: a post-hoc analysis of the DANFLU-1 randomized clinical trial. Clin Microbiol Infect.

[83] Vardeny O., Kim K., Udell J.A. (2021). Effect of high-dose trivalent vs standard-dose quadrivalent influenza vaccine on mortality or cardiopulmonary hospitalization in patients with high-risk cardiovascular Disease. JAMA.

[84] CDC Influenza fact sheet 2024. https://www.cdc.gov/flu/about/keyfacts.htm.

[85] Lane A., Quach H.Q., Ovsyannikova I.G., Kennedy R.B., Ross T.M., Einav T. (2025). Characterizing the short- and long-term temporal dynamics of antibody responses to influenza vaccination. bioRxiv. https://www.medrxiv.org/content/10.1101/2025.02.26.25322965v1.

[86] Tong X., Deng Y., Cizmeci D. (2024). Distinct functional humoral immune responses are induced after live attenuated and inactivated seasonal influenza vaccination. J Immunol.

[87] Sant A.J., Dipiazza A.T., Nayak J.L., Rattan A., Richards K.A. (2018). CD4 T cells in protection from influenza virus: viral antigen specificity and functional potential. Immunol Rev.

[88] Mettelman R.C., Souquette A., Van De Velde L.-A. (2023). Baseline innate and T cell populations are correlates of protection against symptomatic influenza virus infection independent of serology. Nat Immunol.

[89] Shannon I., White C.L., Yang H., Nayak J.L. (2021). Differences in influenza-specific CD4 T-cell mediated immunity following acute infection versus inactivated vaccination in children. J Infect Dis.

[90] Shi W., Wohlwend J., Wu M., Barzilay R. (2024). VaxSeer: selecting influenza vaccines through evolutionary and antigenicity models. bioRxiv. https://www.biorxiv.org/content/10.1101/2023.11.14.567037v3.

