# Supplementary material for: Leveraging pre-vaccination antibody titres across multiple influenza H3N2 variants to forecast the post-vaccination response
Source: eBioMedicine. 2025 May 26;116:105744. doi: 10.1016/j.ebiom.2025.105744 (PMC12155842; doi:10.1016/j.ebiom.2025.105744)
Supplement: Supplementary Figs. S1–S16 and Table S1 [file mmc1.docx]

Supplementary Information for

**Leveraging Pre-Vaccination Antibody Titres across Multiple Influenza H3N2 Variants to Forecast the Post-Vaccination Response**

Stacey et al.

**CONTENT**

**Supplementary Tables**

**Table S1.** Percent of all participants vaccinated two seasons in a row with consistently high (HAI≥80) or consistently low (HAI≤40) titres against the vaccine strain.

**Supplementary Figures**

**Figure S1.** Demographics and null responders in the vaccine studies used to predict responses.

**Figure S2.** Equating analogous viruses across studies.

**Figure S3.** Quantifying the importance of feature combinations.

**Figure S4.** Cross-study predictions are carried out with ≥4 overlapping variants.

**Figure S5.** Predicting post-vac HAI across two datasets.

**Figure S6.** Accuracy of pairwise predictions between vaccine studies.

**Figure S7.** Summary of predictions across datasets.

**Figure S8.** Comparing modelling approaches.

**Figure S9.** Using pre-vaccination titres alone can lead to large prediction error across studies.

**Figure S10.** Null and linear models explain little-to-no variance for recent variants.

**Figure S11.** Comparing within- and cross-study predictions in vaccine studies.

**Figure S12.** Individual virus predictions from Fig 5.

**Figure S13.** Recouping the effects of recent infection history and the antibody ceiling.

**Figure S14.** Characterising “robustly strong” and “robustly weak” vaccine responses.

**Figure S15.** Fold-change of variants in Fig 6 as a function of Δ_Peak_.

**Figure S16.** Alternate categorization of strong/weak fold-change responses.

| **Current → Next Season** | **% Subjects High/High or Low/Low Titres  in Both Seasons (*n*)** | **ΔAA_Epitope_ across vaccine strains (amino acids)** | **ΔAA_Total_ across vaccine strains (amino acids)** |
| --- | --- | --- | --- |
| 2016 → 2017 | 83 (*n*=102) | 0 | 0 |
| 2017 → 2018 | 88 (*n*=149) | 3 | 6 |
| 2018 → 2019 | 73 (*n*=161) | 10 | 16 |
| 2019 → 2020 | 64 (*n*=316) | 11 | 18 |
| 2020 → 2021 | 74 (*n*=252) | 11 | 16 |
| 2021 → 2022 | 84 (*n*=19) | 8 | 10 |
| 2022 → 2023 | 100 (*n*=3) | 0 | 0 |

**Table S1. Percent of all participants vaccinated two seasons in a row with consistently high (HAI≥80) or consistently low (HAI≤40) titres against the vaccine strain.** The right-most column represents the total number of differences in either HA epitopes A-E (ΔAA_Epitope_) or the entire HA (ΔAA_Total_) between the H3N2 vaccine strain’s hemagglutinin (HA) between both seasons. Rows where >80% of subjects showed consistent responses are shaded in grey. The H3N2 vaccine strains are given in **Fig S1A**; note that the H3N2 vaccine strain did not change between the 2016-2017 or 2022-2023 seasons and minimally changed in 2017-2018. The final two rows are based on the 2022/2023 UGA vaccine studies introduced in this work and have far fewer subjects.


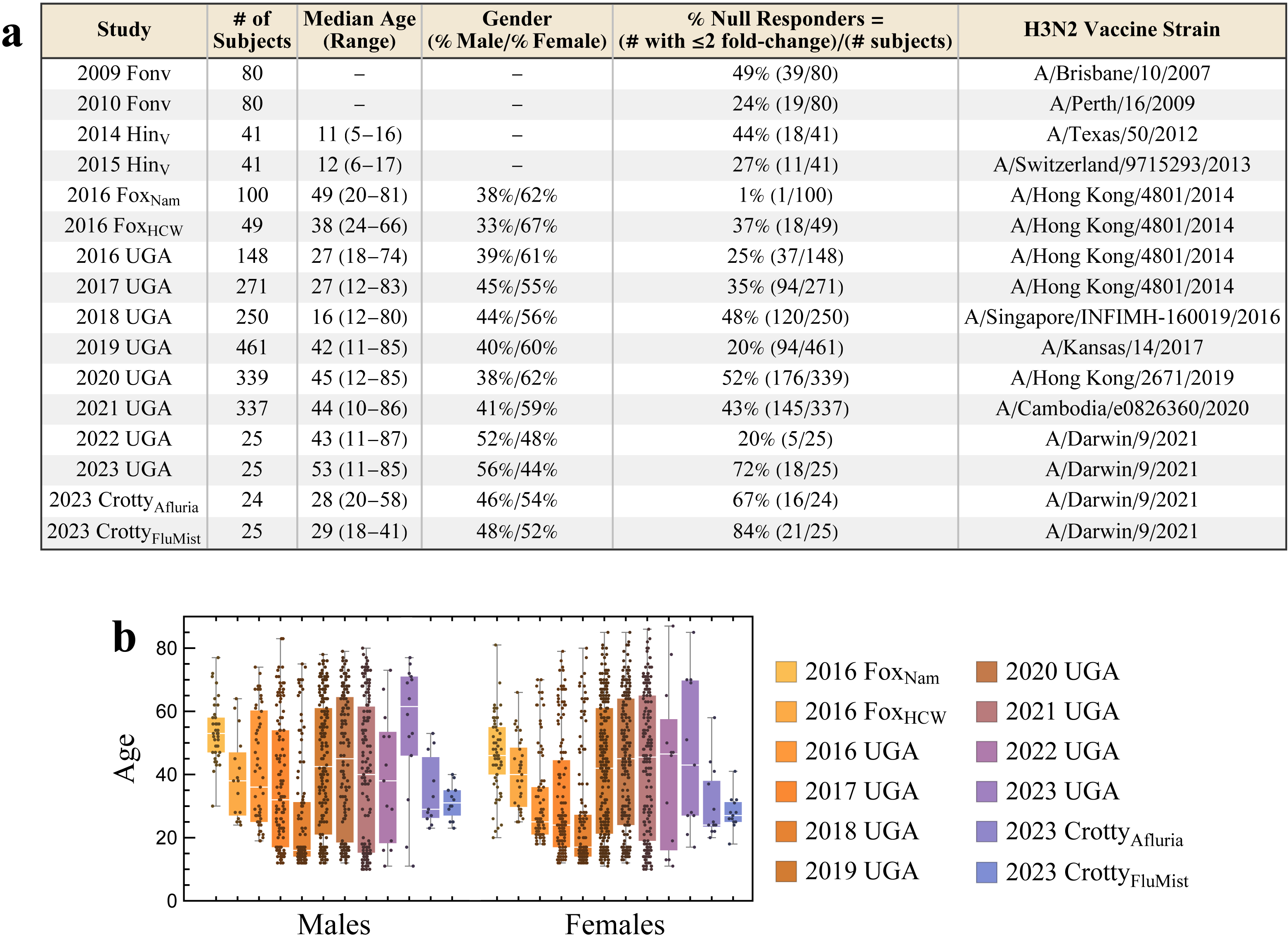


**Figure S1. Demographics and null responders in the vaccine studies used to predict responses.** (a) Null responders are defined as participants whose response 1-month post-vac differed by ≤2-fold across all variants measured. The final four entries represent the new studies introduced in this work; each study enrolled 25 participants, although one participant in the 2023 Crotty_Afluria_ study did not have post-vac measurements because their serum tube clotted during the blood draw. Dashes (–) represent features that were not reported. (b) Age distribution for all males and females (when measured) in each study. For each box plot, the horizontal line indicates the median, the box indicates the interquartile range, and the whiskers indicate 1.5 times the interquartile range.


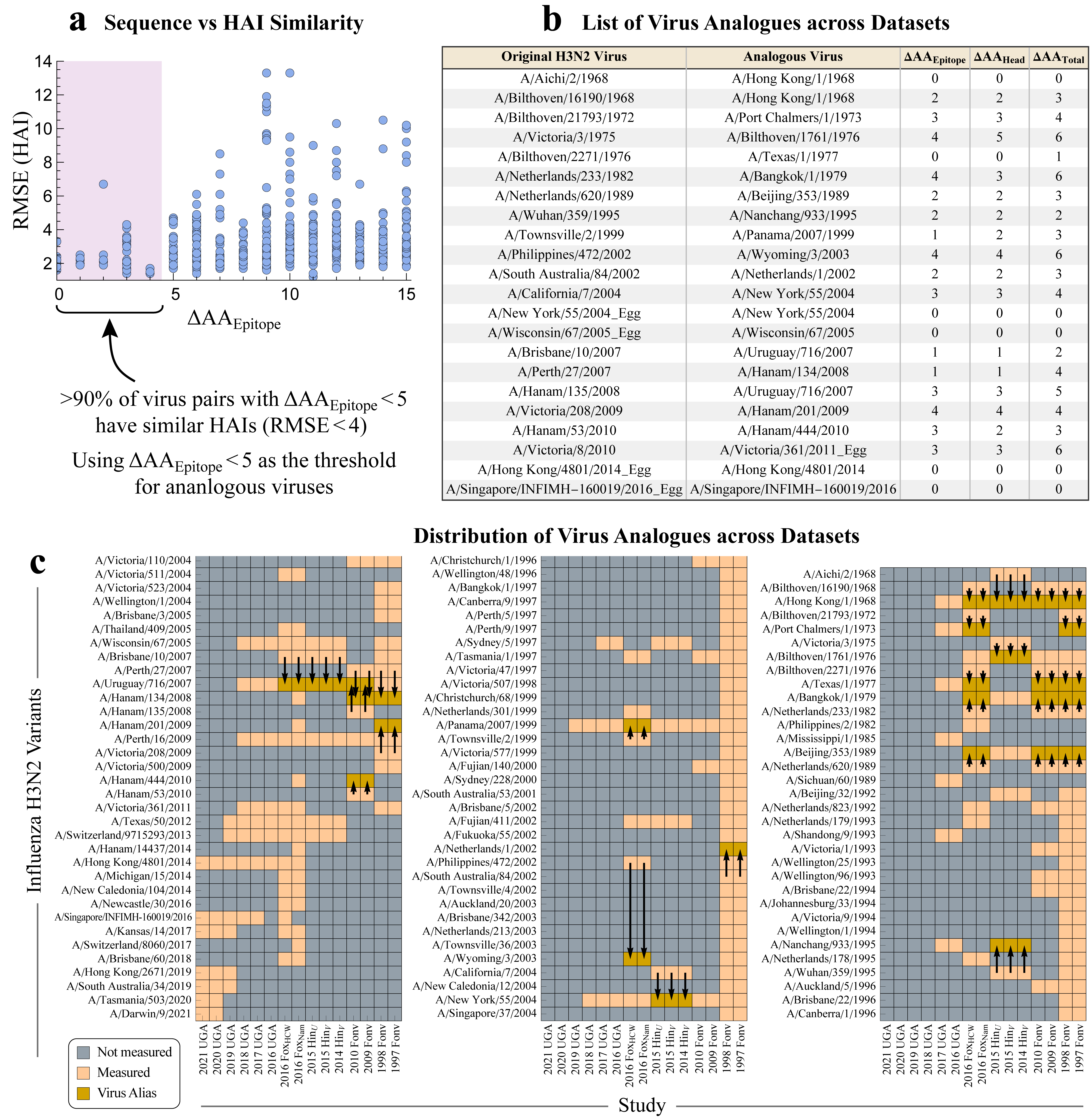


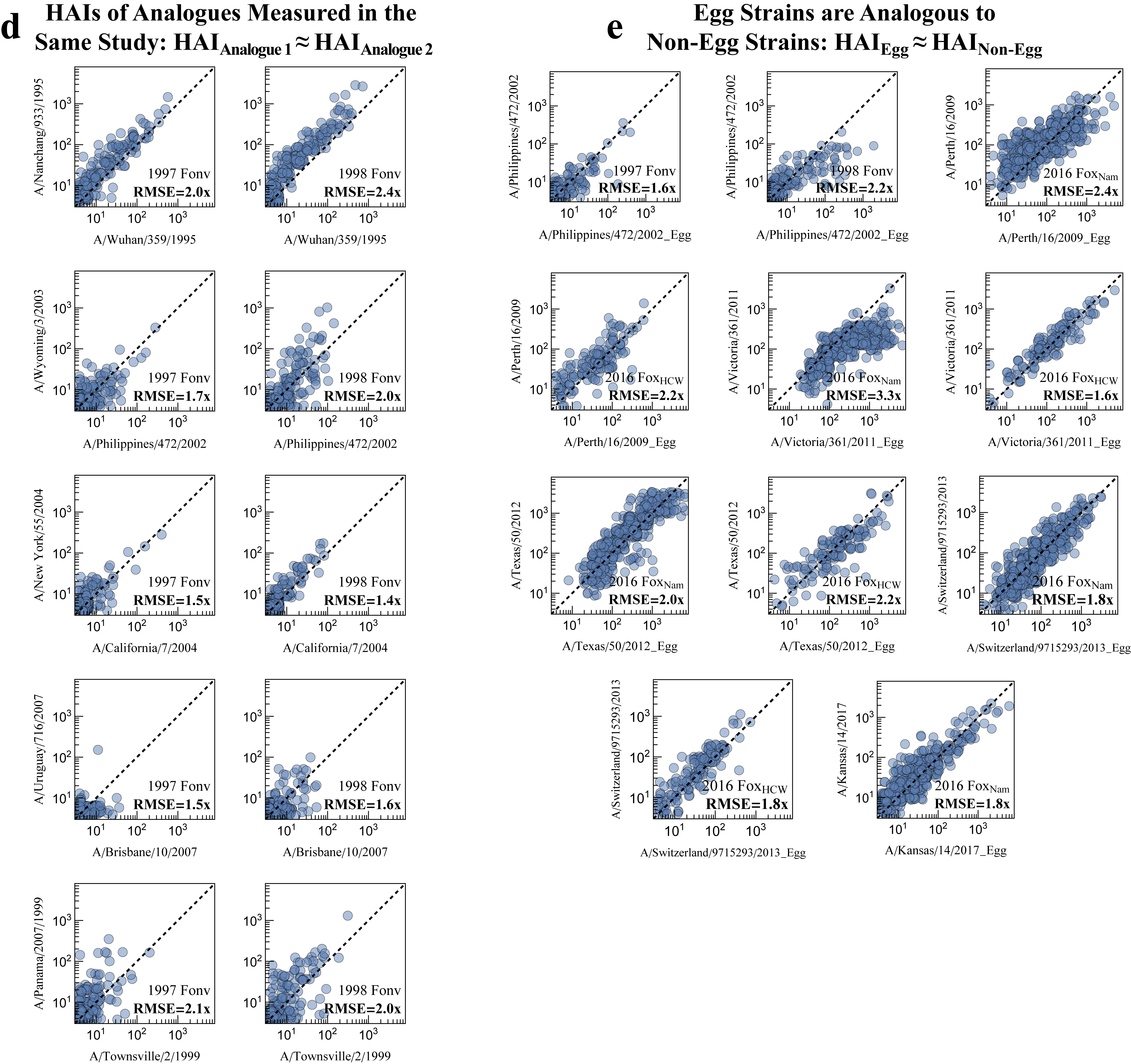


**Figure S2. Equating analogous viruses across studies.** (a) Determining HAI similarity as a function of amino acid mutations across H3N2 epitopes A-E (ΔAA_Epitope_). Within each dataset, for all pairs of viruses with a given ΔAA_Epitope_ [*x*-axis], we computed the root-mean-squared error between their log(HAI) [*y*-axis]. Virus sequences were taken from each study when available and otherwise from GISAID consensus sequences. (b) Using the threshold ΔAA_Epitope_<5 to equate viruses across studies; viruses were equated whenever it increased the number of predictions (by creating more squares in the same row in Panel c). Differences in the HA amino acid sequence are shown considering H3N2 epitopes A-E [ΔAA_Epitope_], the full HA head [ΔAA_Head_], or the full HA head+stem [ΔAA_Total_] between the original variant and its analogue. Most (but not all) epitopes are within the HA head, so ΔAA_Epitope_≤ΔAA_Head_ is generally true. Both ΔAA_Epitope_≤ΔAA_Total_ and ΔAA_Epitope_≤ΔAA_Head_ always hold. (c) List of variants measured in each study, with arrows representing a shift to an analogous strain to increase virus overlap. All egg- and cell-passaged strains were equated, since in every study when both were measured their HAI titres were approximately identical [Panel d]. (d) HAI similarity directly assessed for cases where two virus analogues were measured in the same study. HAIs from analogue 1 (*x*-axis) and analogue 2 (*y*-axis) across all sera and all time points are shown, with most points lying along the diagonal leading to RMSE≲2x. (e) In the vast majority of cases where an egg and non-egg strain were measured in the same study, the HAI of both was nearly identical with an RMSE≲2x.

**
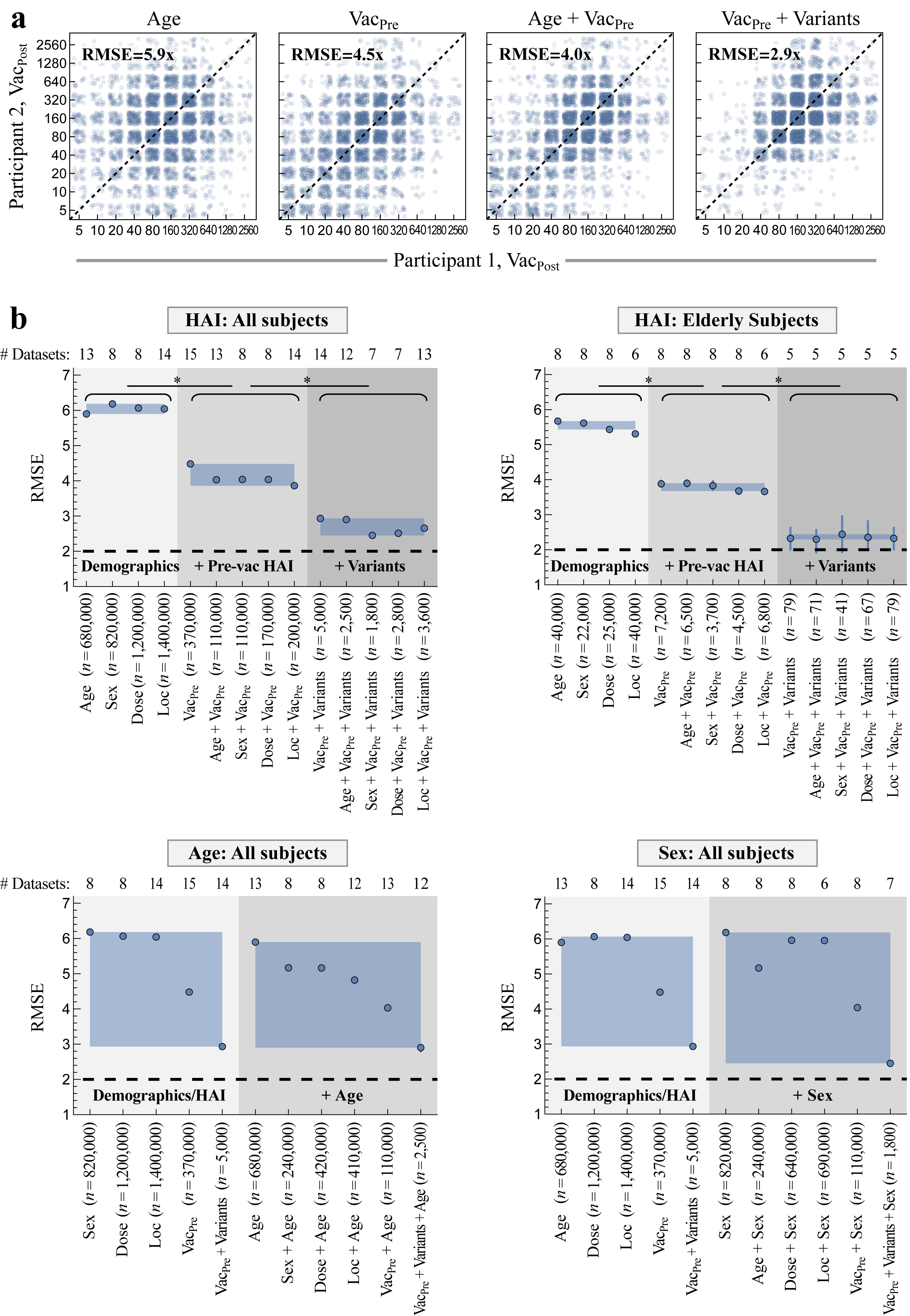
**

**Figure S3. Quantifying the importance of feature combinations.** (a) The vaccine strain’s post-vac HAI (Vac_Post_) across all pairs matching on four potential feature sets, from which the RMSE in **Fig 2** is computed. For clarity, at most 4000 (randomly selected) points are shown, although statistics were computed on all data. Any HAI titre<10 is treated as HAI=5. (b) Analysis from **Fig 2A** quantifying the predictive power of each feature after adding pre-vac HAI against the vaccine strain or variants, age, or sex. Blue rectangles emphasize the span in root-mean-squared error (RMSE, *y*-axis) with/without each feature of interest as emphasized by the different shading. The top-right panel analyses subjects with age≥65 while the other three panels analyse all subjects. 95% CI are only larger than the plot markers for the upper-right panel. The dashed line represents the intrinsic 2-fold error of the HAI assay.^4^ **p*<0.001 in Panels a and b indicate that each feature has a significant change relative to any other feature with a different shading (including non-adjacent shadings) using a one-sided permutation test. No significant change was found in Panels c and d across the two regions with different shading.

**
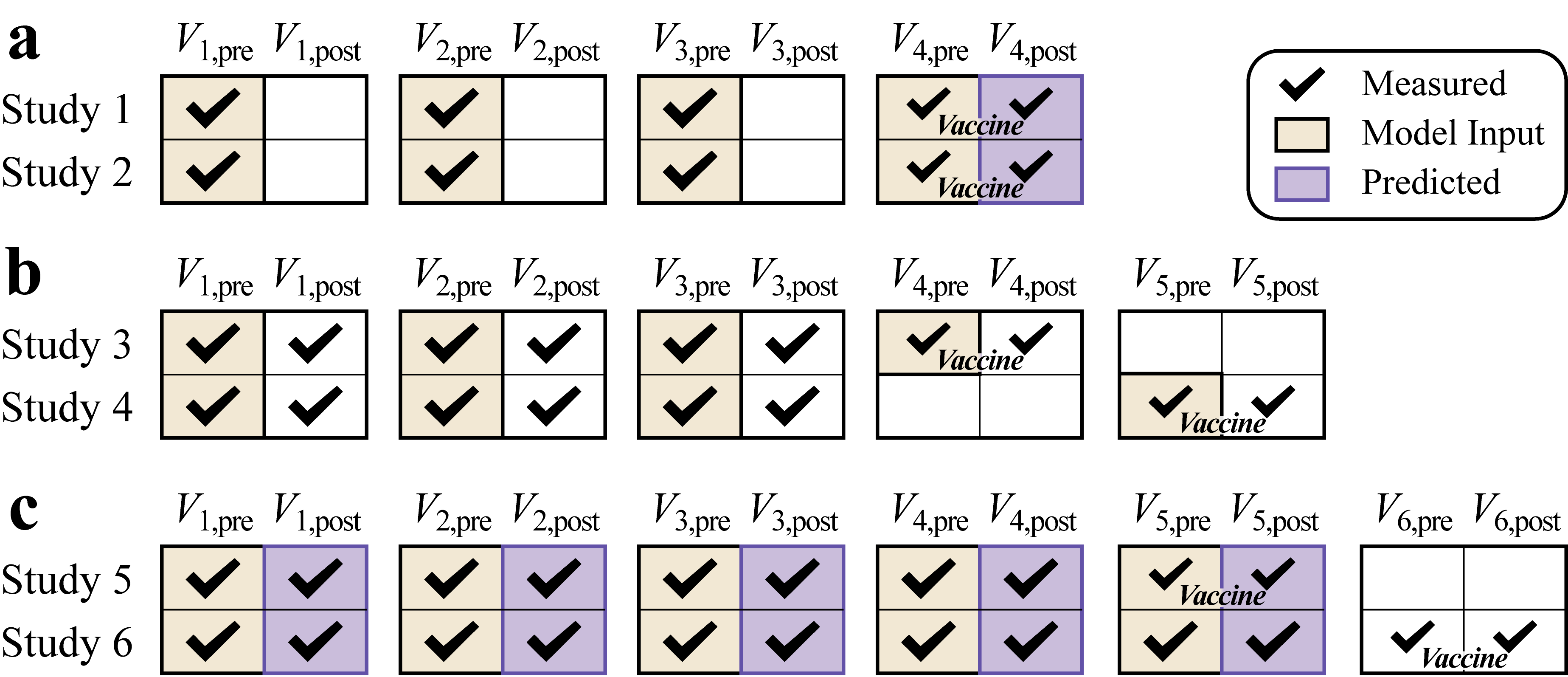
**

**Figure S4. Cross-study predictions are carried out with ≥4 overlapping variants.** (a) Minimal data required to predict *V*_4,post_ in either study. In this work, we only predict across studies administering the same vaccine strain if the studies were carried out in different seasons. (b) No cross-study predictions are possible with <4 overlapping variants. In this case, Study 3 could predict *V*_4,post_ in future studies measuring *V*_1-4,pre_, while Study 4 could predict *V*_5,post_ in future studies measuring *V*_1-3,pre_ and *V*_5,pre_. (c) *V*_1-5,post_ can be predicted in either study, yet we opted to predict from the earlier to the later study. Note that the *V*_5,post_ model learned in Study 5 predicts *V*_5,post_ in Study 6, even though the vaccine strain has changed.

*
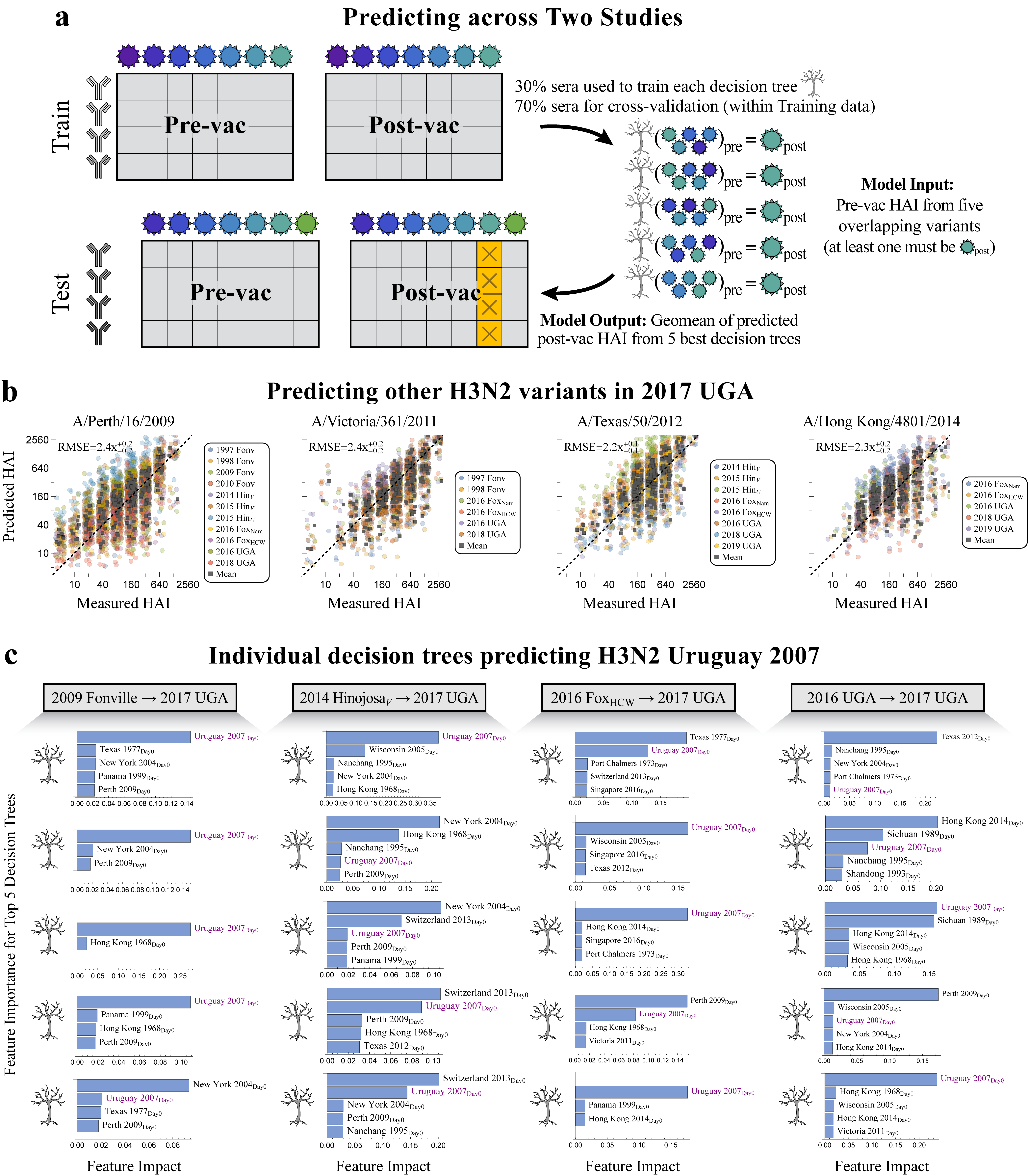
*

**Figure S5. Predicting post-vac HAI across two datasets.** (a) Schematic of the prediction pipeline for one variant (teal) present in two datasets. Decision trees are trained in the upper dataset, using pre-vac HAI to predict the teal variant’s post-vac HAI, and then the top trees (with the lowest cross-validation error in the training dataset) are applied to the lower dataset. This process can be used to predict any of the overlapping variants measured in both datasets (*i.e.*, all but the leftmost variant in the upper dataset or the rightmost variant in the lower dataset). (b) Predictions [analogous to **Fig 3B**] for other representative variants in 2017 UGA. (c) Feature importance when predicting Uruguay 2007’s post-vac HAI [**Fig 3B**] using 2009 Fonville, 2014 Hinojosa*_V_*, 2016 Fox_HCW_, or 2016 UGA. In each case, decision trees were trained using Day 0 HAI from different variants to predict Uruguay 2007’s HAI 1-month post-vaccination. The geometric mean of predictions from the top 5 decision trees (with the lowest internal RMSE) were used to predict Uruguay 2007’s HAI in 2017 UGA. Plots show the absolute value of SHAP feature importance.


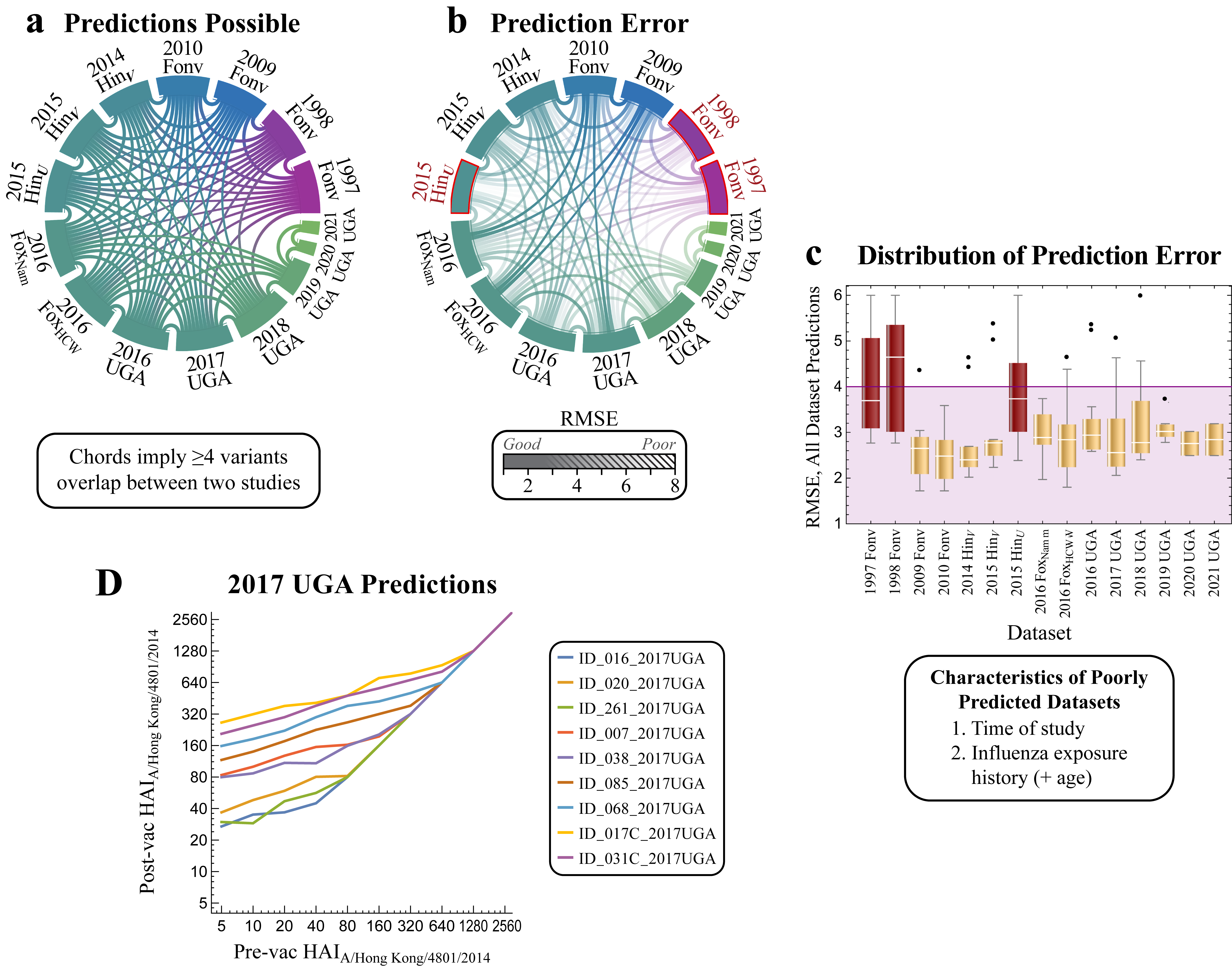


**Figure S6. Accuracy of pairwise predictions between vaccine studies.** (a) Predictions between two studies were carried out when ≥4 variants were measured in both studies, as indicated by each chord. For clarity, studies are coloured from purple-to-green based on the year they were carried out. (b) The error from each pairwise prediction in both directions (mean of Study *S*_1_ predicting Study *S*_2_ and Study *S*_2_ predicting Study *S*_1_) is shown by each chord’s transparency. Highly transparent chords represent poorer predictions (legend shows a transparency gradient on top of a pattern). (c) Distribution of all predictions to and from each study. The three studies boxed in red (Panel b) or shown in red boxes (Panel c) have an upper quartile RMSE>4x (above the purple region), indicating that some features of their study design led to fundamentally different vaccine responses. The characteristics of these three studies are shown below Panel c. (d) Example predictions in 2017 UGA of the vaccine strain H3N2 A/Hong Kong/4801/2014. Post-vac HAI is predicted as a function of pre-vac HAI for nine representative subjects (individual curves). Each subject’s pre-vac HAI against all other variants influences the response. The post-vac HAI is always constrained to be at least as large as the pre-vac HAI, and hence responses always lie above the diagonal (*y*=*x*). For pre-vac HAI≥1280, all predictions lie on this diagonal.

*
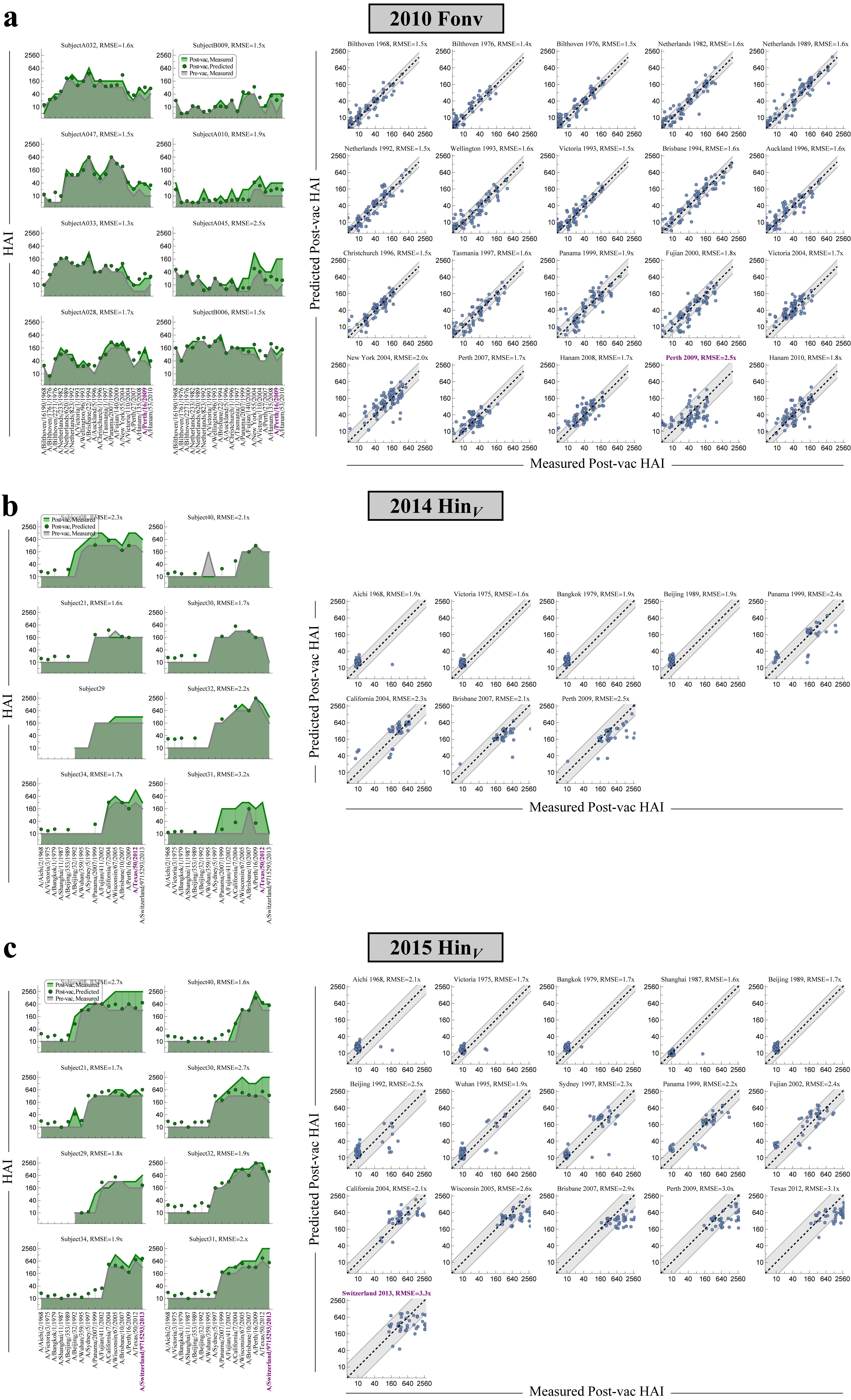
*

**
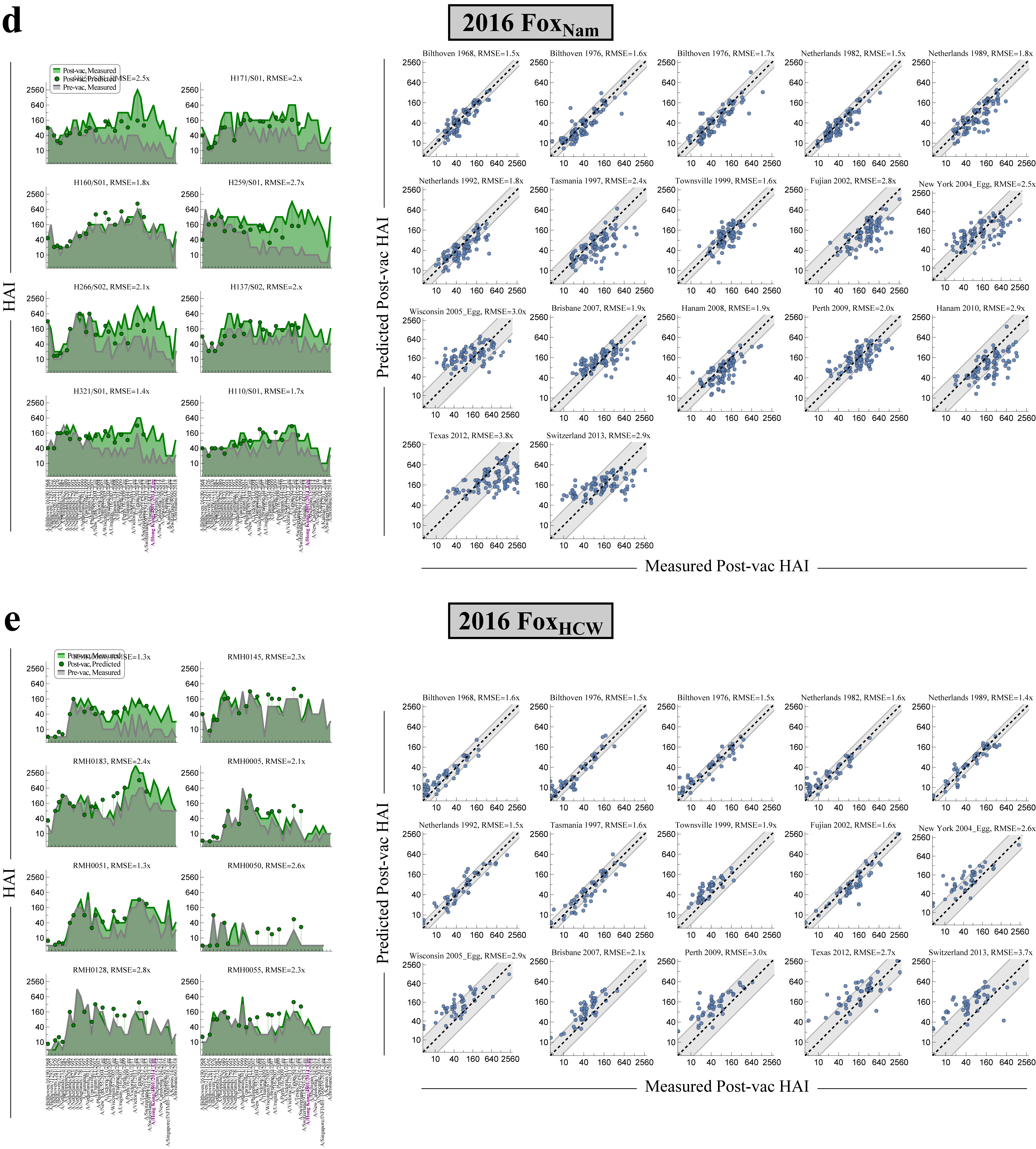
**

**
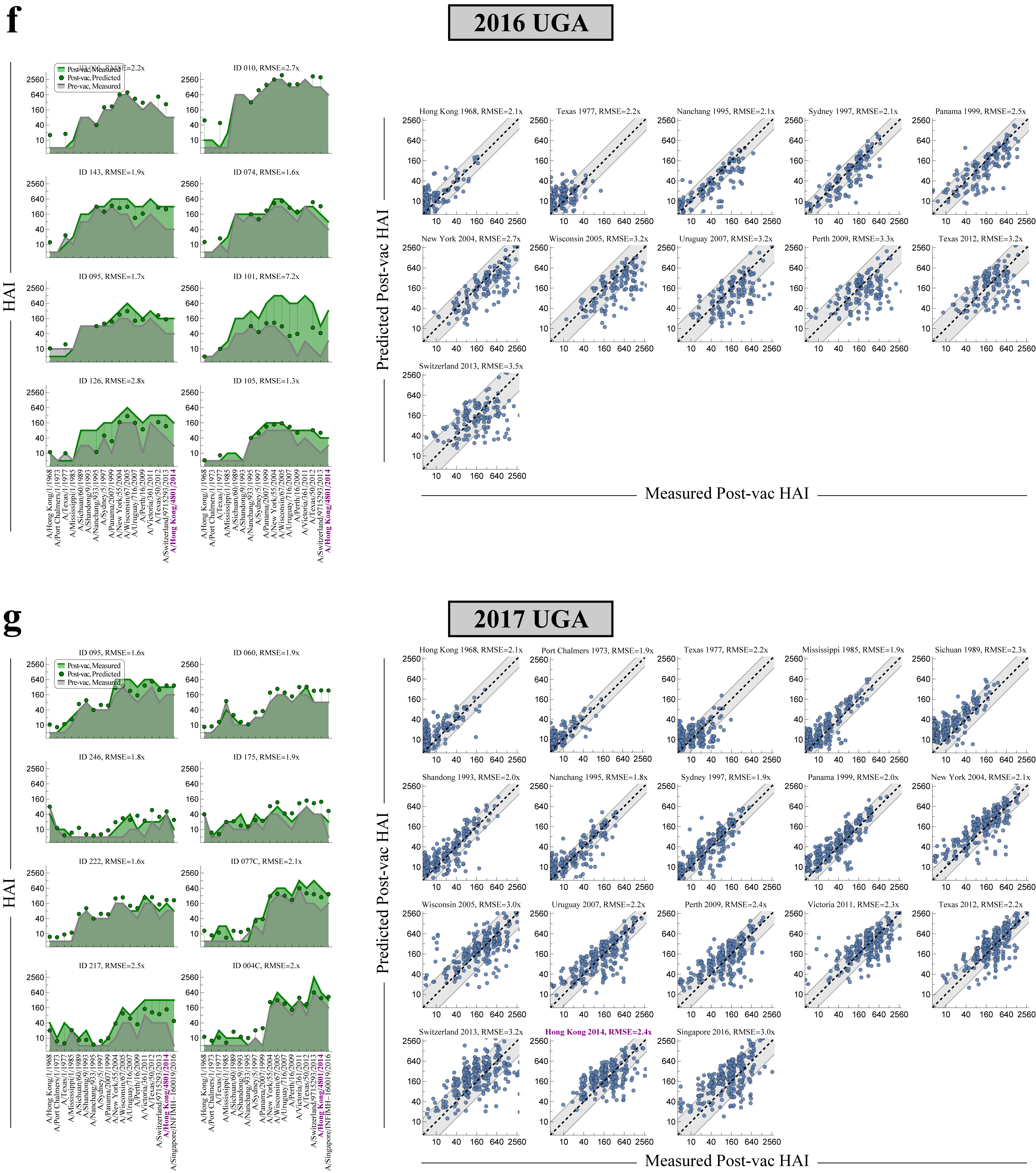
**

**
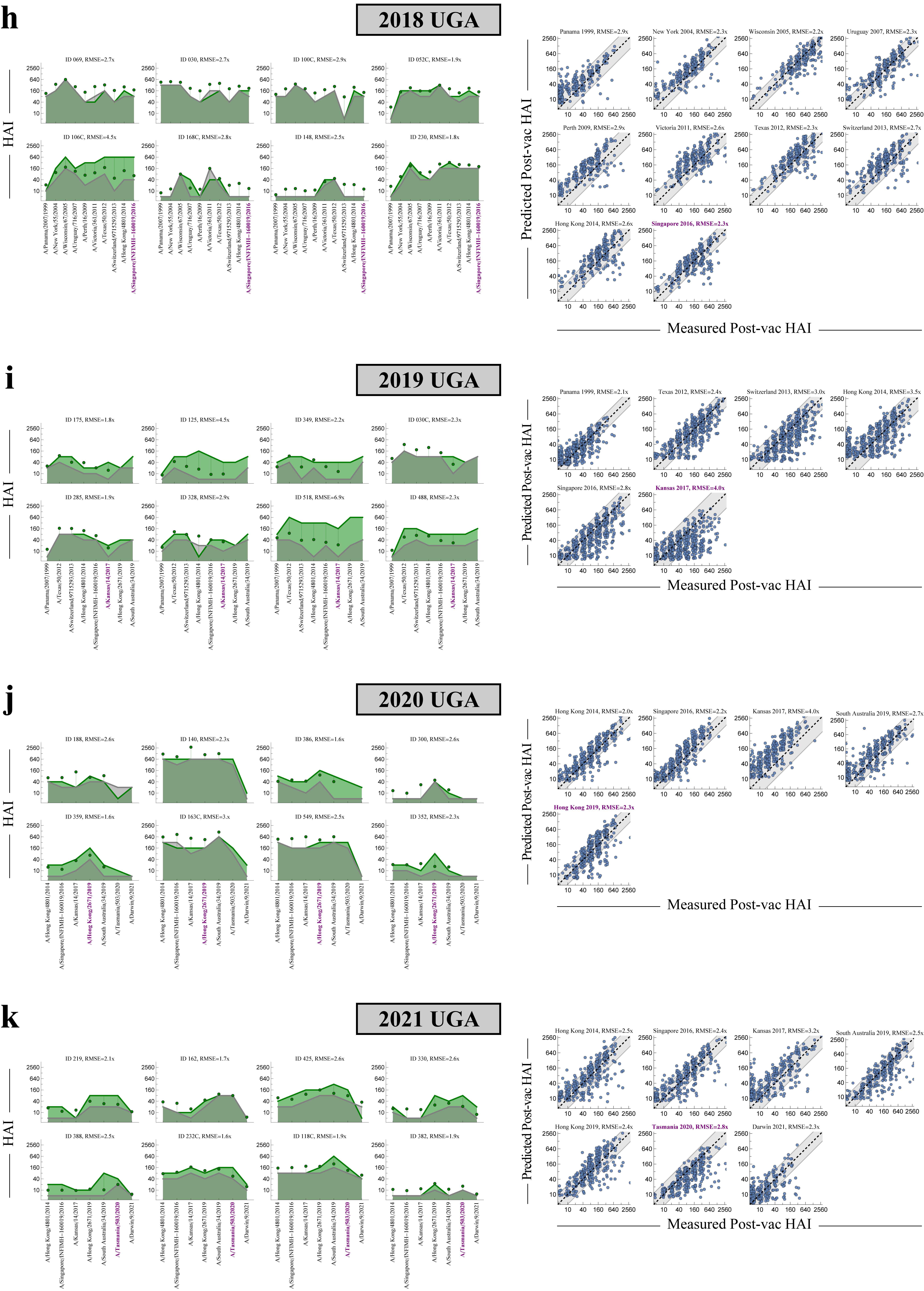
**

**Figure S7. Summary of predictions across datasets.** (a-k) Examples of post-vac predictions for eight randomly chosen subjects [*left*] and up to 20 variants [*right*]. The vaccine strain is highlighted in purple in each study. Some variants may not have predictions if they were not measured in any previous datasets.


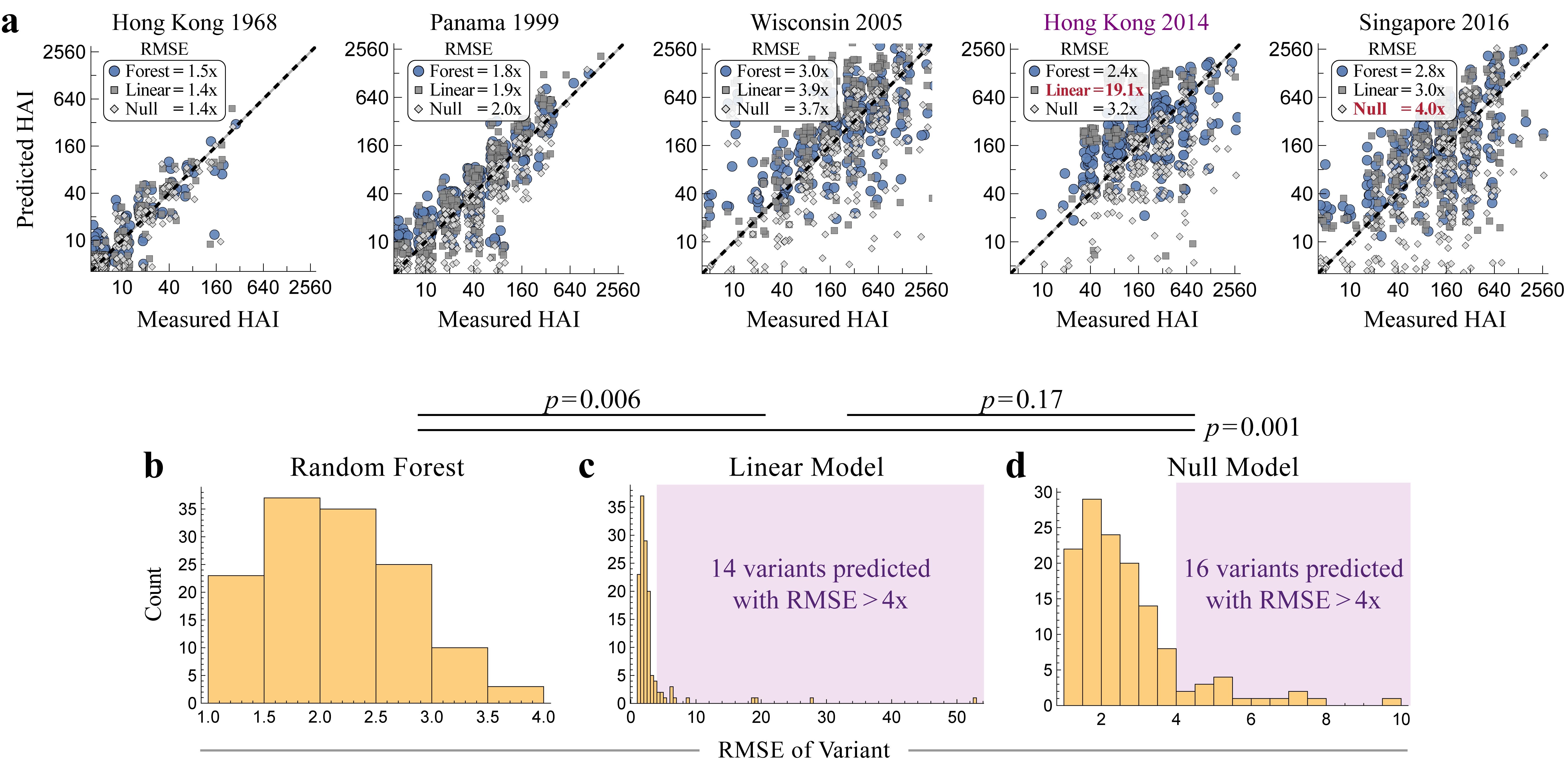


**Figure S8. Comparing modelling approaches.** (a) Predicted versus measured post-vac HAI of five variants in 2017 UGA, showing the random forest predictions (blue, as in **Fig 3F**) overlaid with the linear model (grey squares) and null model (grey diamonds). Cases where RMSE≥4x for the null or linear models is highlighted in red. As in the main text, the vaccine strain H3N2 A/Hong Kong/4801/2014 is shown in purple. (b-d) The distribution of RMSEs for all viruses predicted in each study. (b) The random forest approach described in **Fig 3**. (c) A linear model predicting each variant *V* by training on all (HAI*_V_*_,pre_, HAI*_V_*_,post_) titres from prior datasets. (d) A null model assuming that each variant’s post-vac HAI equals its pre-vac HAI. The number of variants predicted with RMSE>4x is emphasized in each panel. *P* values are calculated using a one-sided permutation test.


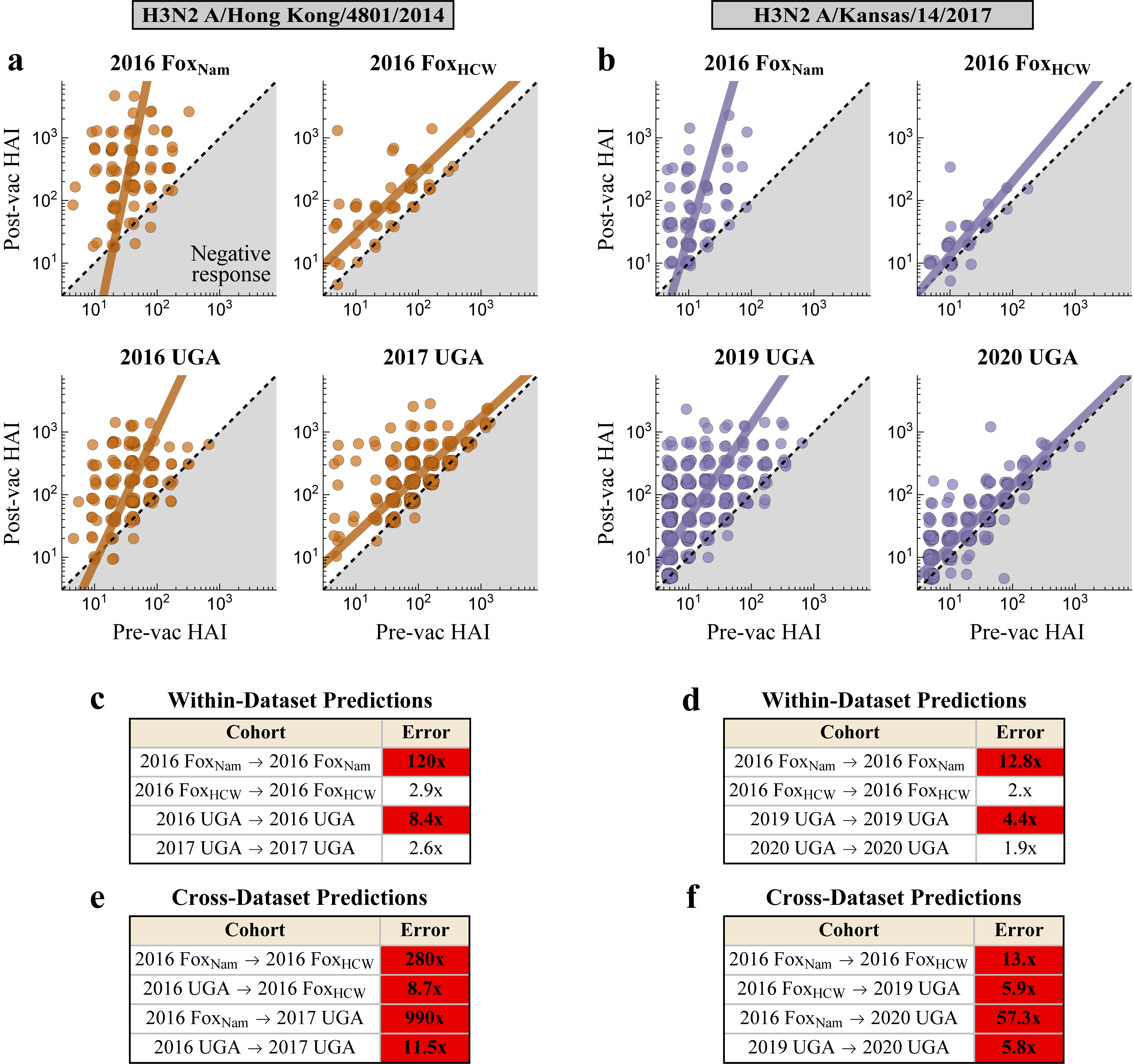


**Figure S9. Using pre-vaccination titres alone can lead to large prediction error across studies.** Comparison of H3N2 A/Hong Kong/4801/2014 [*left*] and H3N2 A/Kansas/14/2017 [*right*] across different studies. (a,b) Pre- versus post-vac HAI titres. The diagonal line represents the null model, while the grey triangle on the bottom-right represents unlikely responses where titres decrease post-vaccination. There is heterogeneity both within one study (distribution of points around each best-fit line) as well as heterogeneity across studies (differences between the best-fit lines). (c,d) For within-dataset predictions of the vaccine strain’s pre→post-vac HAI, linear regression was fit to 70% of the data to predict the remaining 30%, with error showing the mean fold-change across 10 iterations. (e,f) For cross-dataset predictions, all data from one cohort was used to predict all data in the other cohort. Cases where error>4x are highlighted in red. Note that some errors reach >100x.


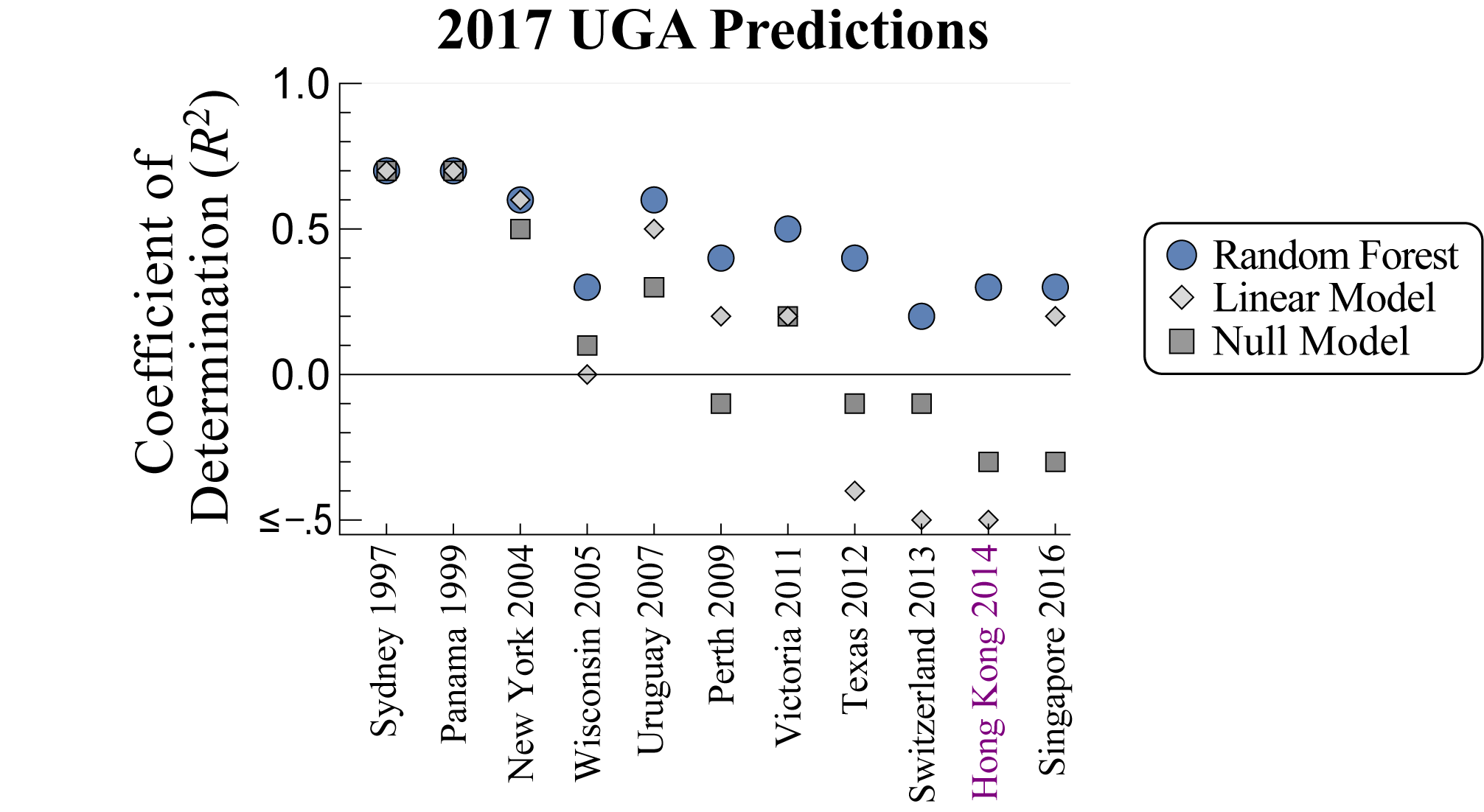


**Figure S10.** **Null and linear models explain little-to-no variance for recent variants.** The coefficient of determination *R*^2^ for variants in 2017 UGA comparing the random forest approach (blue points) with a linear model (grey squares) or null model (grey diamonds).


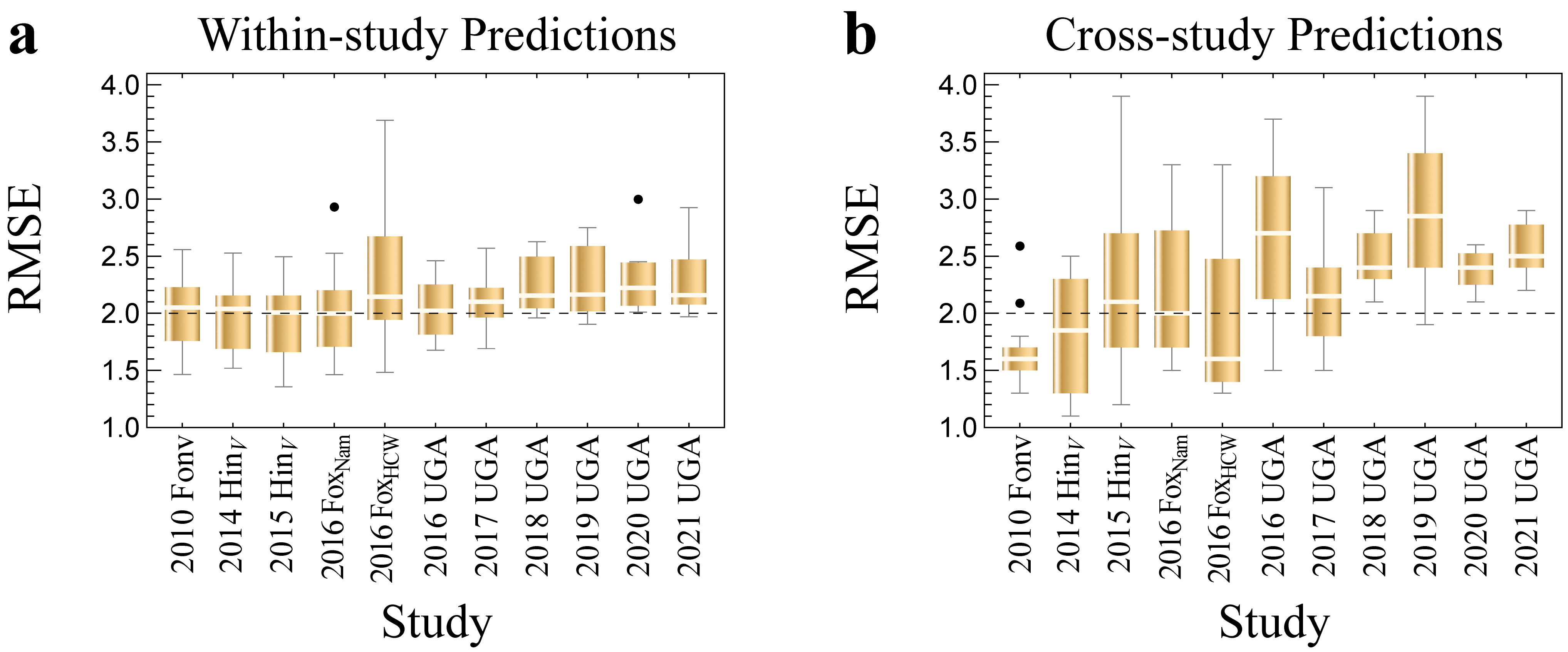


**Figure S11. Comparing within- and cross-study predictions in vaccine studies.** (a) Using 30% of subjects in each study to predict the remaining 70% of subjects in this same study. (b) Reproduction of **Fig 4A** showing the cross-study predictions forward in time carried out in this work.


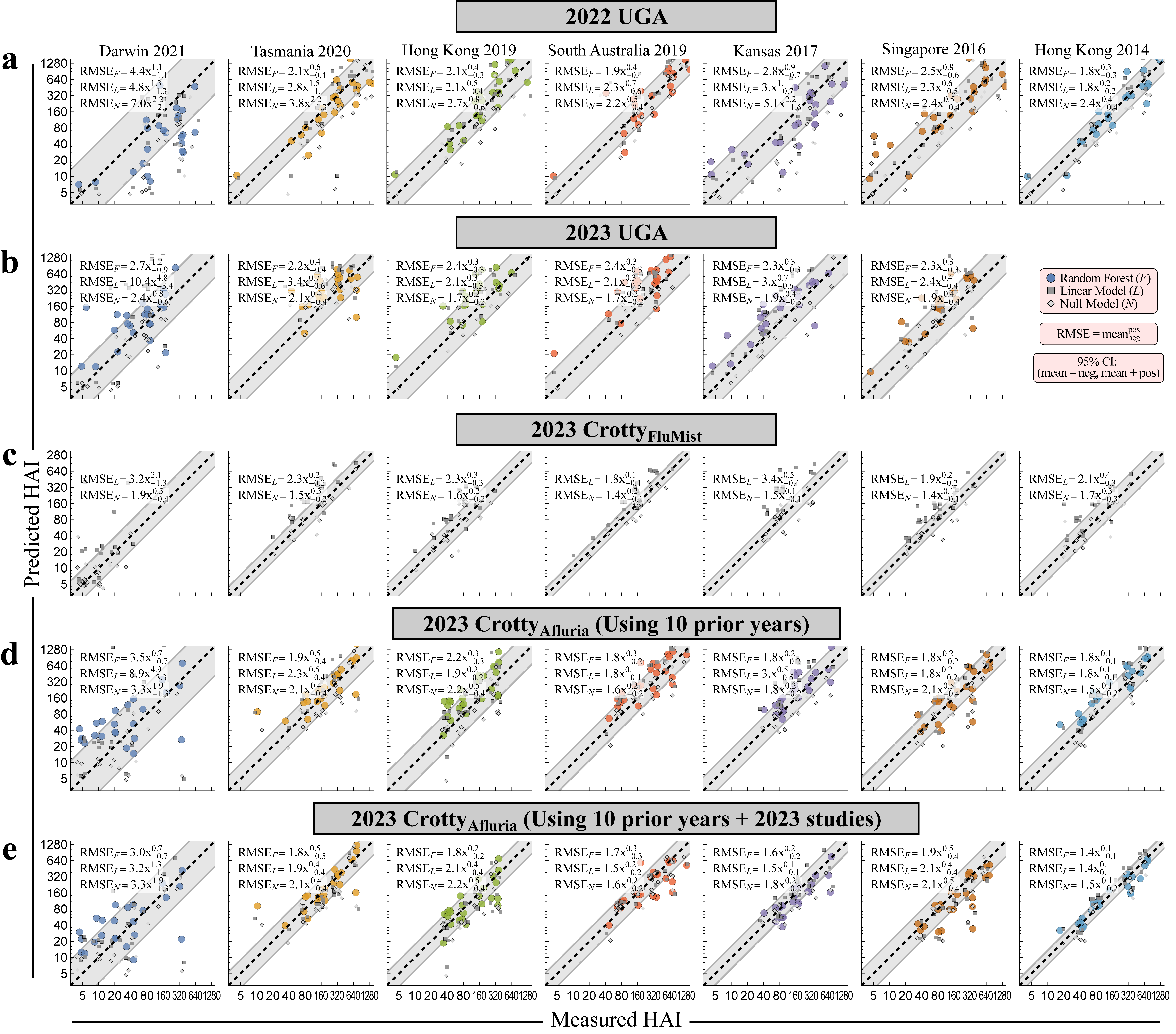


**Figure S12. Individual virus predictions from Fig 5.** Predicted versus measured post-vac HAI for the new vaccine studies (a) 2022 UGA, (b) 2023 UGA [which did not measure Hong Kong 2014], (c) 2023 Crotty_FluMist_, and (d) 2023 Crotty_Afluria_ using the random forest approach (*F*, coloured points), linear model (*L*, grey squares), or null model (*N*, grey diamonds). For all predictions, training was carried out on studies from the past 10 years; random forests were not used for the live attenuated vaccine study (2023 Crotty_FluMist_). RMSE and 95% CI (sub/superscript) are shown in the top-left. (e) Since the 2023 Crotty_Afluria_ study was conducted three months after the 2023 UGA study, an additional specialised prediction was created by finding the best combination of vaccine studies that predicted the 2023 UGA data and then using those to predict 2023 Crotty_Afluria_. The resulting predictions were based upon 2022 and 2023 vaccine studies [some currently unpublished]. The grey diagonal band denotes the width of RMSE*_F_* in panels a, b, d, e and RMSE*_N_* in panel c.


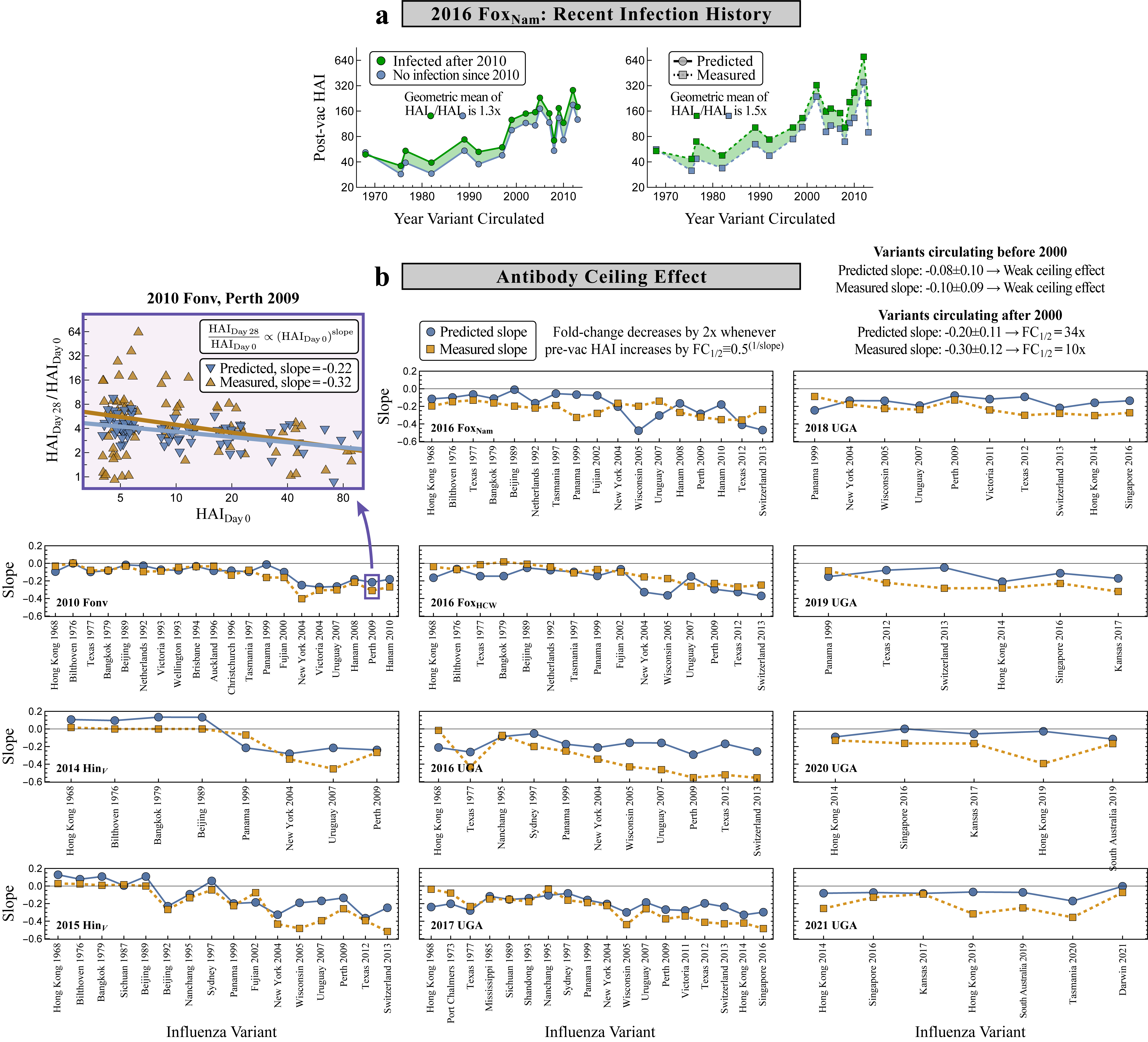


**Figure S13. Recouping the effects of recent infection history and the antibody ceiling.** (a) Predicted versus measured post-vac HAI for the 2016 Fox_Nam_ study, showing the geometric mean titres for *n*=51 individuals with a known influenza H3N2 infection after 2010 (green) and *n*=49 individuals with last confirmed H3N2 infection before 2010 (blue). The shaded area indicates that the more recently infected cohort has a larger HAI. (b) The antibody ceiling effect was computed for each variant and every study using the slope of the linear fit of pre-vac HAI versus fold-change (both on log-axes). Resulting slopes are shown for all measurements and predictions.


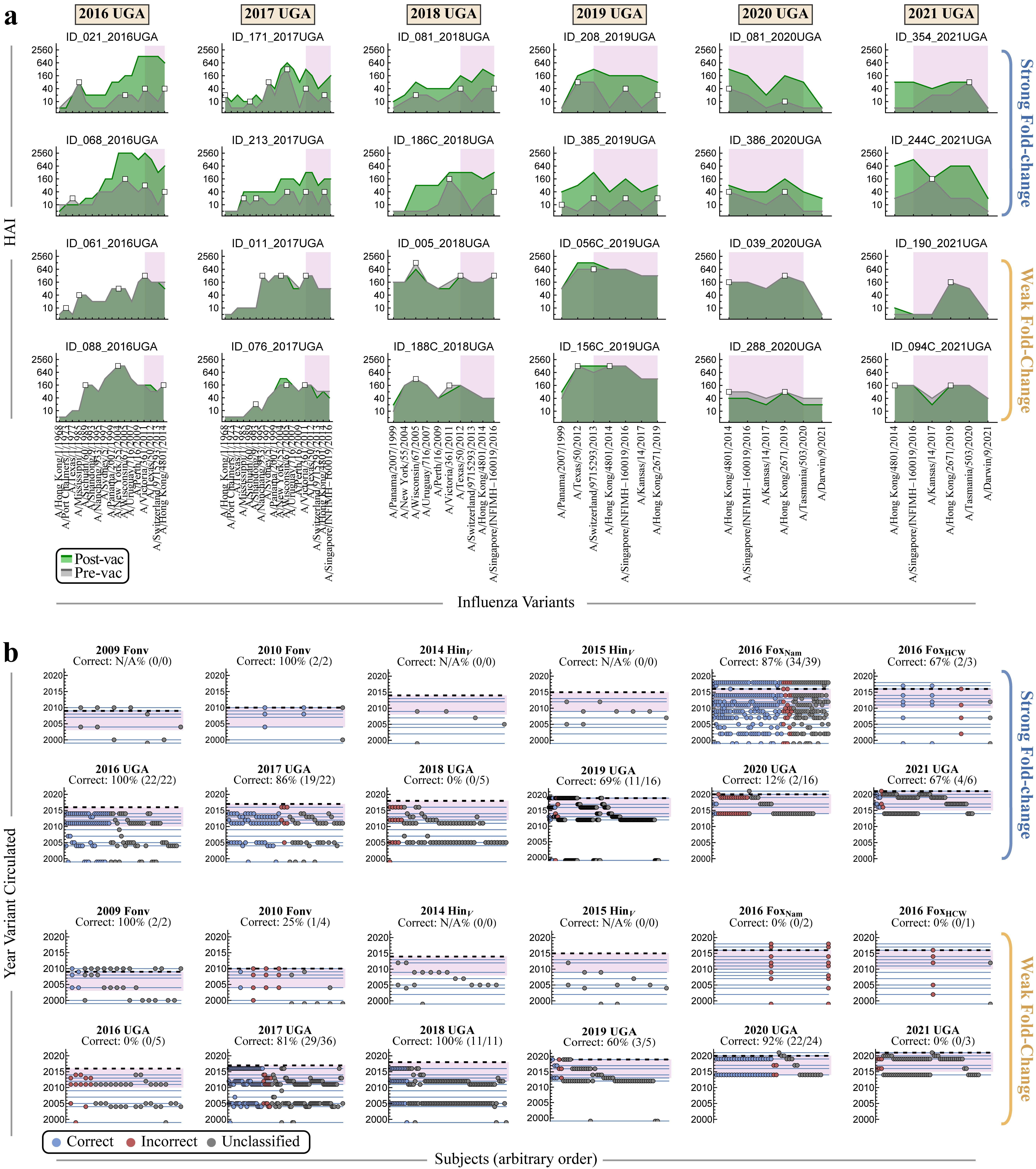


**Figure S14. Characterising “robustly strong” and “robustly weak” vaccine responses.** (a) Representative examples of robust strong or weak responses from the UGA studies. Vaccine responses were defined as strong fold-change (FC≡post-vac GMT/pre-vac GMT≥2.5) or weak fold-change (FC≤1.1) across all variants. Responses were robustly strong/weak if a support vector machine (using a sigmoid kernel) and nearest-neighbours classified each state, as well as ≥75% of perturbed states (varying any single HAI by 2x or 1/2x), as also having this same strong/weak fold-change. (b) Classifying subjects with strong or weak fold-change post-vaccination from all studies. Each subject is either correctly classified (blue), incorrectly classified (red), or not classified (grey). Blue lines represent the years of all variants from 2000 onwards in each study, and points on each column represent the peaks of an individual’s HAI profile. In Panels a and b, the purple region represents the region in which Δ_Peak_ is computed (*i.e.*, the period 0-6 years before each study). The dashed line in Panel b represents the year of each study.


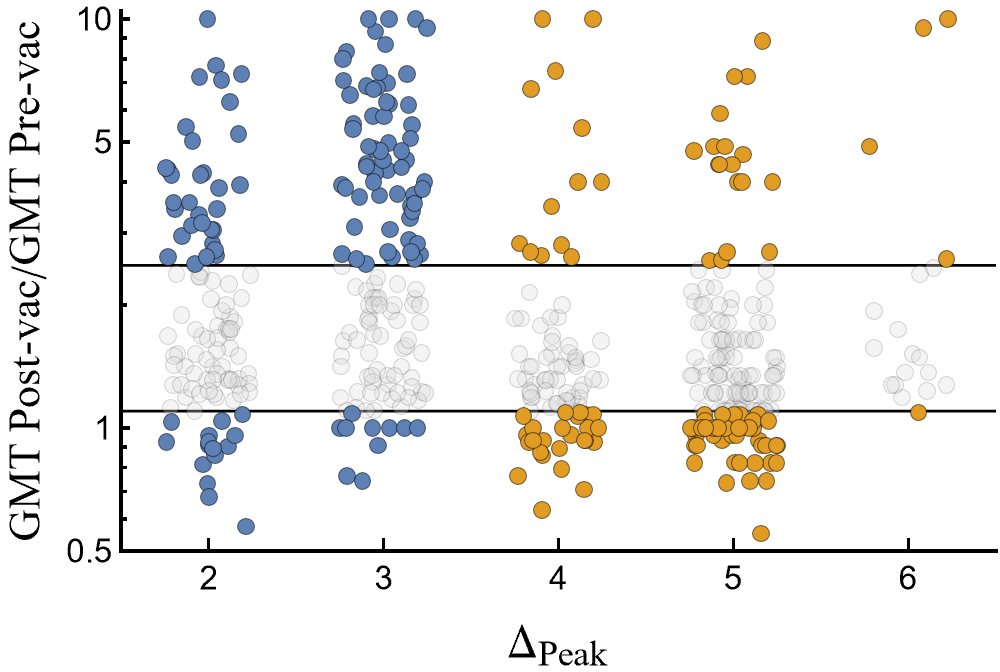


**Figure S15. Fold-change of variants in Fig 6** **as a function of Δ_Peak_.** Individuals were predicted to have strong fold-change (FC≡post-vac GMT/pre-vac GMT≥2.5 across all variants) if 2≤Δ_Peak_≤3 [blue] and weak fold-change (FC≤1.1) if 4≤Δ_Peak_≤6 [gold]. Individual with an intermediate fold-change (1.1≤FC≤2.5) were not considered.


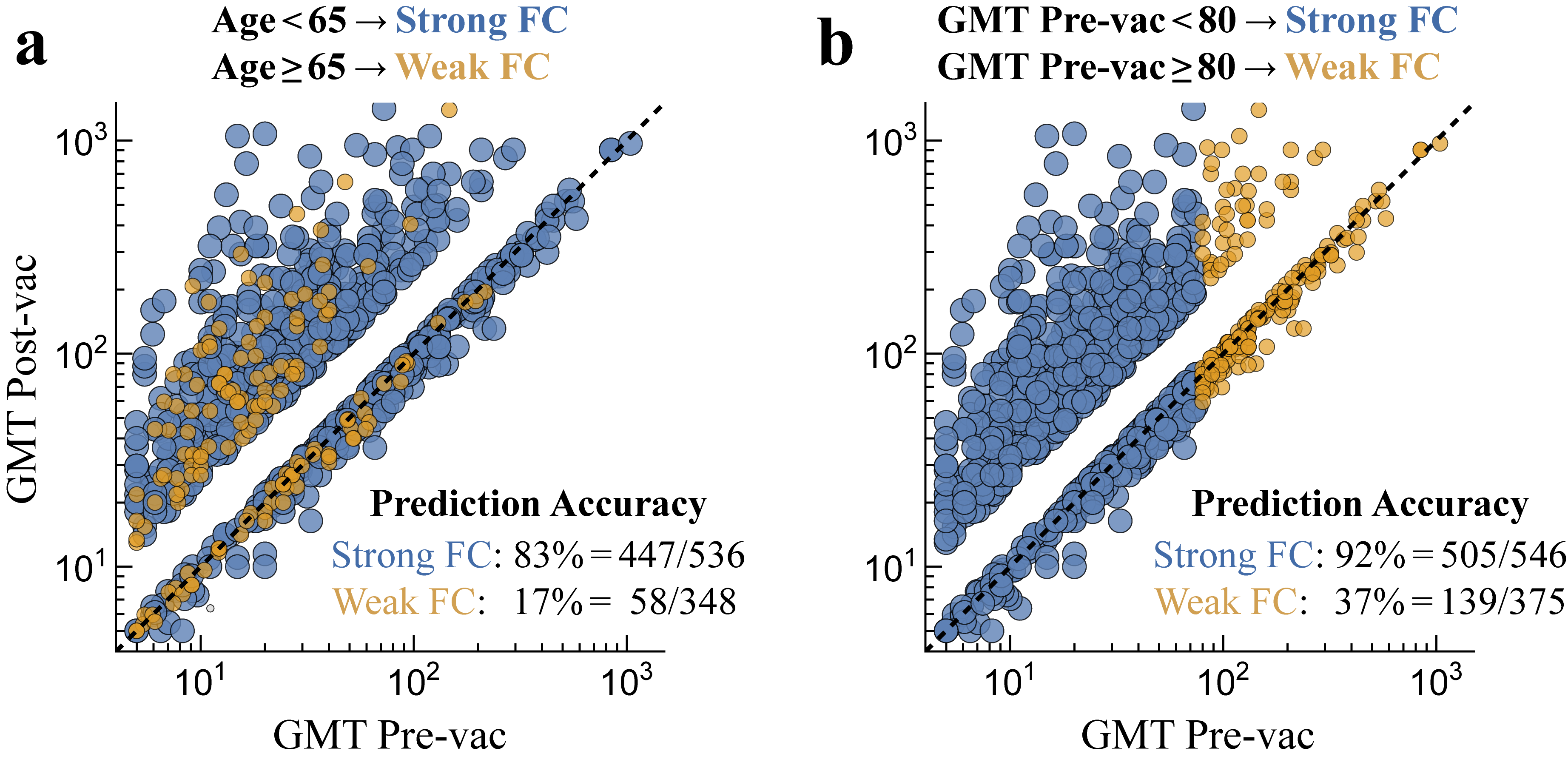


**Figure S16. Alternate categorization of strong/weak fold-change responses.** (a) An age-based categorization assuming the elderly (age≥65) exhibit weak fold-change (FC) post-vaccination. (b) Pre-vac GMT-based categorization assuming that individuals that start with higher titres (GMT pre-vac≥80) will exhibit weak fold-change. In both panels, statistics in the bottom-right show how many of the actual strong responses (FC≥2.5) or weak responses (FC≤1.1) were correctly predicted.
